# Early life microbial exposures shape the *Crassostrea gigas* immune system for lifelong and intergenerational disease protection

**DOI:** 10.1186/s40168-022-01280-5

**Published:** 2022-06-04

**Authors:** Manon Fallet, Caroline Montagnani, Bruno Petton, Luc Dantan, Julien de Lorgeril, Sébastien Comarmond, Cristian Chaparro, Eve Toulza, Simon Boitard, Jean-Michel Escoubas, Agnès Vergnes, Jacqueline Le Grand, Ingo Bulla, Yannick Gueguen, Jérémie Vidal-Dupiol, Christoph Grunau, Guillaume Mitta, Céline Cosseau

**Affiliations:** 1 IHPE, CNRS, Ifremer, Univ. Montpellier, Univ. Perpignan via Domitia, Perpignan, France; 2 Ifremer, UBO CNRS IRD, LEMAR UMR 6539, Argenton, France; 3 Ifremer, IRD, Univ Nouvelle-Calédonie, Univ La Réunion, ENTROPIE, F-98800 Nouméa, Nouvelle-Calédonie France; 4 CBGP, CIRAD, INRAE, Institut Agro, IRD, Université de Montpellier, Montpellier, France; 5 MARBEC, CNRS, Ifremer, IRD, Univ Montpellier, Sète, France; 6 Ifremer, UMR 241 Écosystèmes Insulaires Océaniens, Labex Corail, Centre Ifremer du Pacifique, BP 49, 98725 Tahiti, French Polynesia

**Keywords:** Oyster, Aquaculture, Microbiota, Innate immune shaping, Epigenetic, DNA methylation

## Abstract

**Background:**

The interaction of organisms with their surrounding microbial communities influences many biological processes, a notable example of which is the shaping of the immune system in early life. In the Pacific oyster, *Crassostrea gigas*, the role of the environmental microbial community on immune system maturation — and, importantly, protection from infectious disease — is still an open question.

**Results:**

Here, we demonstrate that early life microbial exposure durably improves oyster survival when challenged with the pathogen causing Pacific oyster mortality syndrome (POMS), both in the exposed generation and in the subsequent one. Combining microbiota, transcriptomic, genetic, and epigenetic analyses, we show that the microbial exposure induced changes in epigenetic marks and a reprogramming of immune gene expression leading to long-term and intergenerational immune protection against POMS.

**Conclusions:**

We anticipate that this protection likely extends to additional pathogens and may prove to be an important new strategy for safeguarding oyster aquaculture efforts from infectious disease. tag the videobyte/videoabstract in this section

Video Abstract

**Supplementary Information:**

The online version contains supplementary material available at 10.1186/s40168-022-01280-5.

## Background

Interactions of hosts with their associated and surrounding microbial communities can have deep implications for host fitness [1–3]. Notably, the natural microbial environment contributes to the maturation of the immune system and to the establishment of mechanisms for pathogen recognition and protection. Disruption of balanced host-microbiota interactions results in various immune and systemic disorders [4–7]. In vertebrates, many studies have emphasized the critical role of microbial colonization during early developmental stages to durably imprint the immune system [5, 8, 9]. This early life biological embedding predicts that exposure to nonpathogenic microorganisms or their metabolites can reprogram the threshold and function of innate immune responses [8, 10, 11] to confer increased and persistent immunocompetence, echoing the emerging concept of “trained immunity.” This concept proposes adaptive properties of innate host-defense mechanisms, whereby innate immunity can retain “memory” of earlier challenges, enabling a more efficient response and increased survival capacity to subsequent pathogen assaults [12–15]. While many studies have examined the molecular mechanisms that support the trained immunity in the mammalian context, especially the implication of epigenetic-based events, little is known about how these ideas may extend to the less specific and systemic impact resulting from the microbiota interaction.

The present study focuses on the Pacific oyster *Crassostrea gigas*, which represents one of the most important marine invertebrate aquaculture species in the world. As filter feeders, oysters interact with a rich microbial environment composed of commensal and pathogenic microorganisms that continuously challenge their immune system [16–18]. *C. gigas* immune system is set up early during the development [19, 20], and this raises the question of the role played by these surrounding microbial communities during early development on oyster physiology and immunity. Despite the lack of memory lymphocytes, *C. gigas* possesses potent immune cells called hemocytes which are able to induce efficient innate immune responses based on highly conserved immune features among which the NF-κB and IFN-like pathways [21]. Recent studies have also shown that oyster immune system can be stimulated to improve their immune response toward bacterial or viral pathogens [22, 23]. Oysters exposed to killed *Vibrio* bacteria exhibit a stronger immune response at cellular and molecular levels promoting an enhanced hemocyte phagocytosis and cell regeneration upon secondary infection with live bacteria [22, 24]. In addition, oyster stimulation with a viral mimic (poly(I:C)) induces an efficient long-term and sustainable antiviral response mainly carried by IFN-like pathways which improves the subsequent resistance and survival of oysters during a viral infection by OsHV-1. Interestingly, this improvement could be maintained across generations [25].

*C. gigas* suffers mass mortalities that affect juvenile stages, decimating up to 100% of young oysters in French farms. In recent years, this mortality syndrome, called Pacific oyster mortality syndrome (POMS), has become panzootic, being observed in all coastal regions of France and numerous other countries worldwide [26]. POMS is a polymicrobial and multifactorial disease, with biotic and abiotic factors influencing the disease outbreak [27]. The central role of a herpes-like virus, OsHV-1-μvar, in POMS has been demonstrated; viral infection triggers an immune-compromised state that induces microbiota dysbiosis and subsequent bacteremia caused by opportunistic bacteria, ultimately leading to oyster death [28].

Recent reviews have suggested that environmental manipulation could be used to produce a desired phenotype and could be applied to critical issues in aquaculture [29–31]. Others have highlighted the potential of hologenomics for application in animal production [32]. Furthermore, it has been recently reported that [33–35] organisms are much more sensitive to environmental cues during early stages of development rather than in adulthood or later stages in life [33–35]. In the context of these emerging insights, we raised the question whether a nonpathogenic environmental microbiota exposure during *C. gigas* early larval development could shape the immune system to change their susceptibility to an infectious disease like POMS. We found that oyster lineages that were exposed to a microorganism-enriched environment in early life had a markedly increased survival rate when challenged with POMS in later life as well as in the subsequent generation. Concomitantly, we sampled these oysters and characterized their bacterial microbiota, transcriptomic response, and genetic and epigenetic profiles. We showed that the microbial exposure caused a significant and long-lasting shift in the oysters’ resident microbiota and strongly modified the expression of immune-related and metabolic genes. We further identified epigenetic signatures that may underlie the durable effect of the early life microbial exposure. These findings open new avenues for the development of microbiome-targeted prophylactic approaches to mitigate diseases of invertebrates of economic importance.

## Methods

### Zootechnics and production of the two oyster generations

**Fig. 1:**
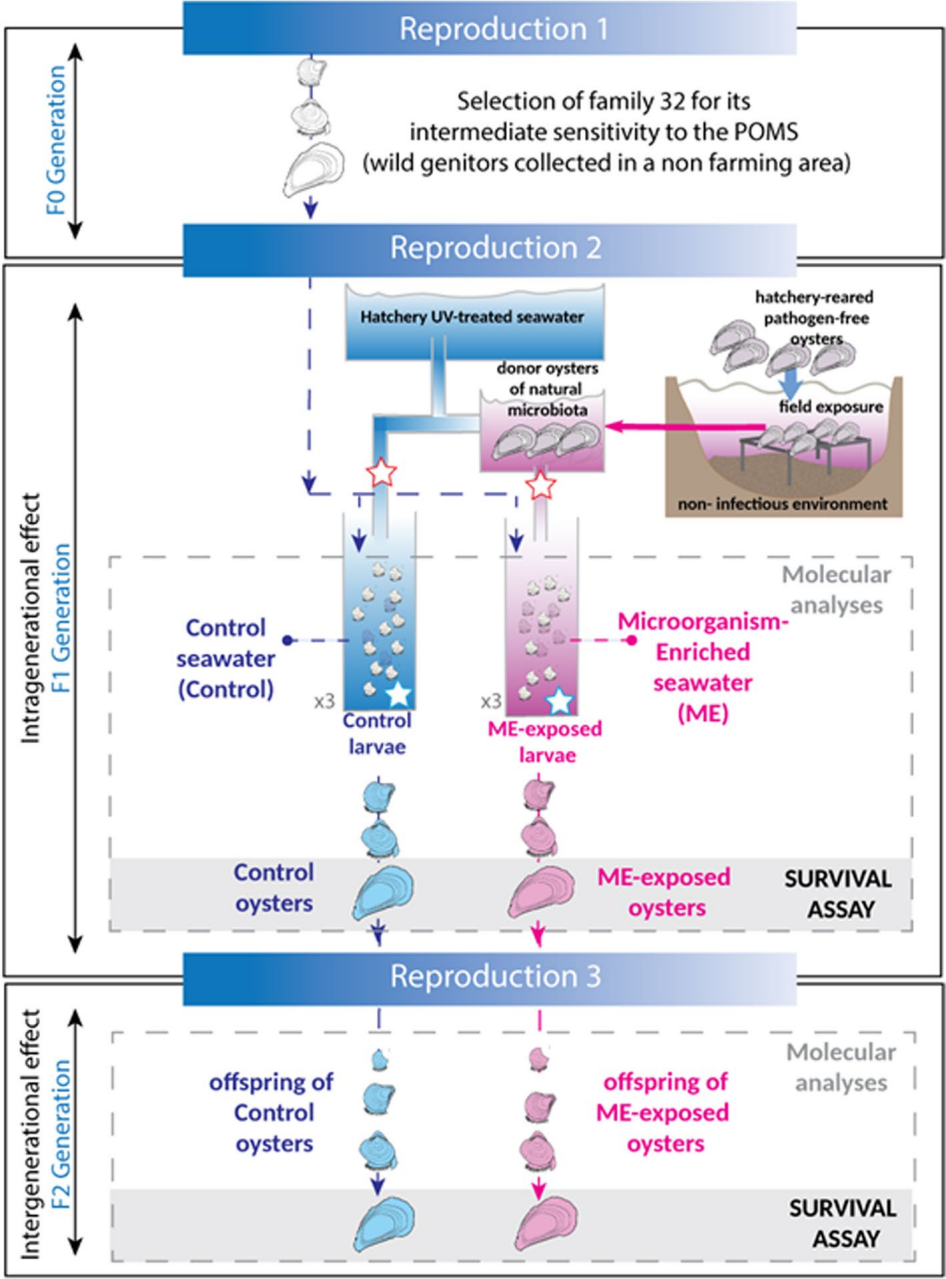
Overall experimental design for larval microbial exposure and reproduction of *C. gigas*. Biparental reproduction was performed to generate the family 32 (selected during the ANR decipher project, see [28]). The family 32 was chosen for its intermediate susceptibility to the POMS disease. The F1 generation was generated in March 2016 by full-sibmultiparental reproduction. Just after fertilization, the oyster larvae were exposed to a hatchery environment (filtered and UV-treated control seawater = control seawater, blue color) or to a natural microbe-enriched environment (microorganism-enriched seawater = ME seawater, pink color). The microorganisms used to enrich the seawater came from donor oysters that acquired their microbiota in the field during a POMS-free period (T° < 16 °C, no OsHV-1). The donor oysters were transferred from the field site to the hatchery and placed upstream of the breeding pipes (3 replicates per condition) in order to transmit their microbiota to recipient larvae via seawater flux. This exposure started 2 h after F0 gametes fecundation and lasted for 10 days. During exposure, donor oysters were renewed three times. This experimental design aimed at mimicking the microorganism-rich seawater that is met in natural environment. After 10 days, both ME-exposed and control oysters were raised in standard hatchery conditions (filtered and UV-treated hatchery seawater). Additionally, both control and ME-exposed oysters were used to perform multiparental reproduction and generate the F2 generation. The F2 progeny was raised in standard conditions with control seawater. Samples were taken all along the life of the F1 and F2 oysters for molecular biology analyses (see Additional file 1: Table 2) and for a pathogen challenge assay at juvenile stages (survival assay, 120 days after fertilization). Red stars indicate seawater sampling for 16S amplicon sequencing analysis, and blue stars indicate seawater sampling for cultivable bacterial analysis

Oyster reproduction was conducted at the Ifremer facility (Argenton and Bouin, France) at bio-secured conditions by filtration and irradiation of seawater as previously described [28, 36]. The F0 generation has been produced from a biparental reproduction with one male and one female of the same geographical origin (Fig. 1). Among 15 families generated and analyzed during a previous project [28], family 32 (Fa.32) was chosen for its intermediate sensitivity to the disease (56% of cumulative mortality during Atlantic experimental infection). Its genitors were collected in the delta of the “Vidourle” river (lat 43.553906-long-4.095175) in a non-farming area meaning that they have not passed through the selective filter due to the infectious environment met in farming area. In March 2016, adults were used to generate the F1 generation by multiparental reproduction. The number of genitors (approximately 100) used for each reproduction and fertilization success is shown in Table 1 of Additional file 1. Fertilization was performed in 5 L of filtered and UV-treated seawater at 21 °C without renewing the seawater. After 2 h, fecundation rate was recorded (Additional file 1: Table 1), and oyster embryos were transferred into breeding pipes for larval rearing. An open flow system which allows for constant renewal of the seawater was used for optimized larval rearing. At this point, the embryos were separated into two groups (Fig. 1): the microorganism-enriched seawater-exposed group (ME-exposed) in which oysters were exposed to a nonpathogenic natural microbiota right after fecundation for 10 days and the control group (control) in which oyster larvae were raised in filtered and UV-treated seawater. For ME seawater exposure, pathogen-free donor oysters (NSI for “Naissains Standardisés Ifremer” or “standardized spats from Ifremer”) were used as described in Petton et al. [36–38]. These NSI donor oysters were placed in March 2016 in a farming area (“Rade de Brest, Pointe du château,” France, Atlantic Ocean-lat. 48.335263-long-4.317922) during a POMS-free period (water temperature < 16 °C, no mortality registered in the field, https://wwz.ifremer.fr/observatoire_conchylicole/Resultats-par-annee/Resultats-nationaux-2016/Mortalite-par-site-et-par-classe-d-age) allowing them to adopt the microbial environment. At this period of the year, the temperature (14 °C) was below the threshold for disease induction and the NSI donors are expected to be pathogen-free [39, 40]. These healthy NSI donors were then transferred back to the laboratory and placed in tanks upstream of the breeding pipes of the “ME-exposed” F1 larvae. Seawater was flowing from the tank of the donor oysters to the recipient F1 larvae to expose them to the ME seawater. This allowed the transmission of microbiota from donor oysters to recipient larvae via water flow (Fig. 1). This ME-exposed condition was designed to mimic the microbial condition that the oyster larvae face in their surrounding natural environment in nature compared to the hatchery control condition. The exposure lasted for 10 days, and donor oysters were replaced 3 times during that period (batch 1 placed at day 0, batch 2 at day 3, and batch 3 at day 7). Each NSI-donor batch had a total biomass of 1000 g containing individual oysters with a mean single weight of 0.17 g. Following the 10 days of exposure and the rest of their life until next reproduction, both groups (ME-exposed and control oysters) were maintained in control conditions. In March 2017, roughly 80 to 100 genitors were used for each reproduction (numbers and fertilization success are indicated in Table 1 of the Additional file 1). After fertilization, the F2 oysters were all raised in the same standard hatchery conditions. No exposure was performed on this F2 generation. For both F1 and F2 generations, samples were taken throughout the life span of the oysters for omics analyses (see Table 2 of Additional file 1 for details), and a phenotypic assay (survival test) was performed at day 120 when the oysters reached the juvenile stage.

### Seawater quality control

Seawater was collected upstream of the recipient oysters’ breeding pipes for control and ME seawater (see Fig. 1 for water sampling position). A total of 3 L, 2 L, and 0.25 L seawater samples were filtered on 10 μm, 0.8 μm, and 0.2 μm pore size filters, respectively (Whatman™, Nucleopore™ Track-Etch Polycarbonate Membrane, 47-mm filters; ref. 111115 — 10 μm; ref. 111109 — 0.8 μm; ref. 111106—0.2 μm). Filtrates were analyzed by subsequent qPCR analysis targeting the bacterial 16S rDNA gene for total bacterial analysis. For total bacterial cultivable analysis, seawater was collected inside each tank of control and ME seawater, every day during the exposure (see Fig. 1 for water sampling position). A total of 100 μL of subsamples of seawater were spread on marine agar Petri dishes (1:10 dilution) that were incubated at 21 °C for 6 days before counting the number of total bacterial colony-forming units (CFUs).

### Field and ecologically realistic experimental infections

For the F1 and F2 generations, at day 100, ME-exposed and control juveniles and offspring of ME-exposed and control juveniles were brought back from Bouin Ifremer facility to Argenton and placed in controlled environment to be acclimatized 3 weeks before disease induction. On day 120 (Table 3 of the Additional file 1), the juvenile oysters from both ME-exposed and control conditions (or their offspring) were subjected to an ecologically realistic experimental infection (Fig. 1 of the Additional file 1) as described in [28, 36]. The weight of recipient and donor individuals used per condition is indicated in Table 3 of the Additional file 1. During this experimental infection, cumulative mortality was monitored every 12 h for up to 15 days for both donors and recipients for both generations. In parallel, oysters were placed in a farming environment (farming area in “Logonna Daoulas,” lat 48.335263—long−4.317922) during the disease outbreak. As soon as the first mortality appeared in this area, the dynamic of mortality was monitored daily for 3 weeks and then every 2 weeks until the end of September, when seawater temperature is below 16 °C. Two-hundred and 100 individuals per condition were used for field disease monitoring for the F1 and F2 generation, respectively.

### Survival curves

Statistical data analysis on survival data was carried out in (GraphPad Prism for Windows, GraphPad software, La Jolla, USA). Survival rates were represented as Kaplan-Meier curves. Significant differences in survival rates between conditions were evaluated using a log-rank test.

### DNA and RNA extraction

Juvenile oyster pools were ground in liquid nitrogen in 50 ml stainless steel bowls with 20 mm-diameter grinding balls (Retsch MM400 mill). These oyster powders (stored at −80 °C) were then used for RNA and DNA extractions as previously described [28]. Genomic DNA from powdered oyster tissues or pools of 10,000 to 20,000 frozen larvae was extracted with the NucleoSpin Tissue kit from Macherey-Nagel (reference 740952.250) according to the manufacturer’s protocol with an additional step of RNAseA treatment (Macherey-Nagel, cat. #740505). Prior to a 90-min enzymatic lysis, an additional 12 min mechanical lysis (Retsch MM400 mill) was performed with zirconia/silica beads (BioSpec). DNA concentration and purity were checked with a NanoDrop ND-1000 spectrometer (Thermo Scientific) and QuBit 2.0 Fluorometer Invitrogen (Life Technologies Corporation).

Total RNA was extracted from oyster powders (10 mg) or pools of 10,000 to 20,000 frozen larvae. Samples were homogenized in 1500 μl of Tri-Reagent (Zymo Research; ref. R2050-1-200). Prior to extraction, insoluble materials were removed by centrifugation at 12,000 × *g* for 10 min at 4 °C, and supernatant was incubated with 0.2 volumes of chloroform at room temperature for 3 min. After centrifugation at 12,000 × *g* for 15 min at 4 °C, total RNA recovered from the aqueous phase was extracted using the Direct-Zol™ RNA Miniprep kit (Zymo Research; ref. R2052) according to the manufacturer’s protocol. RNA concentration and purity were checked with NanoDrop ND-1000 spectrometer (Thermo Scientific), and its integrity was analyzed by capillary electrophoresis with a BioAnalyzer 2100 (Agilent).

### Bacteria and virus detection and quantification

Detection and quantification of OsHV-1 and total bacteria were performed using quantitative PCR as previously described [28]. For quantification of total bacteria in seawater, we used relative quantification normalized by the volume of filtered seawater (3 L, 2 L, or 0.25 L), and then relative proportions in each fraction (10 μm, 0.8 μm, and 0.2 μm pore size filters) were added together to obtain the overall quantification.

### 16S barcoding analysis

Samples used for microbiota analyses are indicated in Table 2 of the Additional file 1. For each time point, 3 biological replicates were used. For each sample, 16S rDNA amplicon libraries were generated targeting the variable V3V4 loops for bacterial communities [41]. Paired-end sequencing with a 250-bp length was performed at the McGill University (Génome Québec Innovation Centre, Montréal, Canada) for F1 generation and in Perpignan University (platform “bio-environnement,” Perpignan, France) for the F2 generation on a MiSeq instrument (Illumina).

**Fig. 2:**
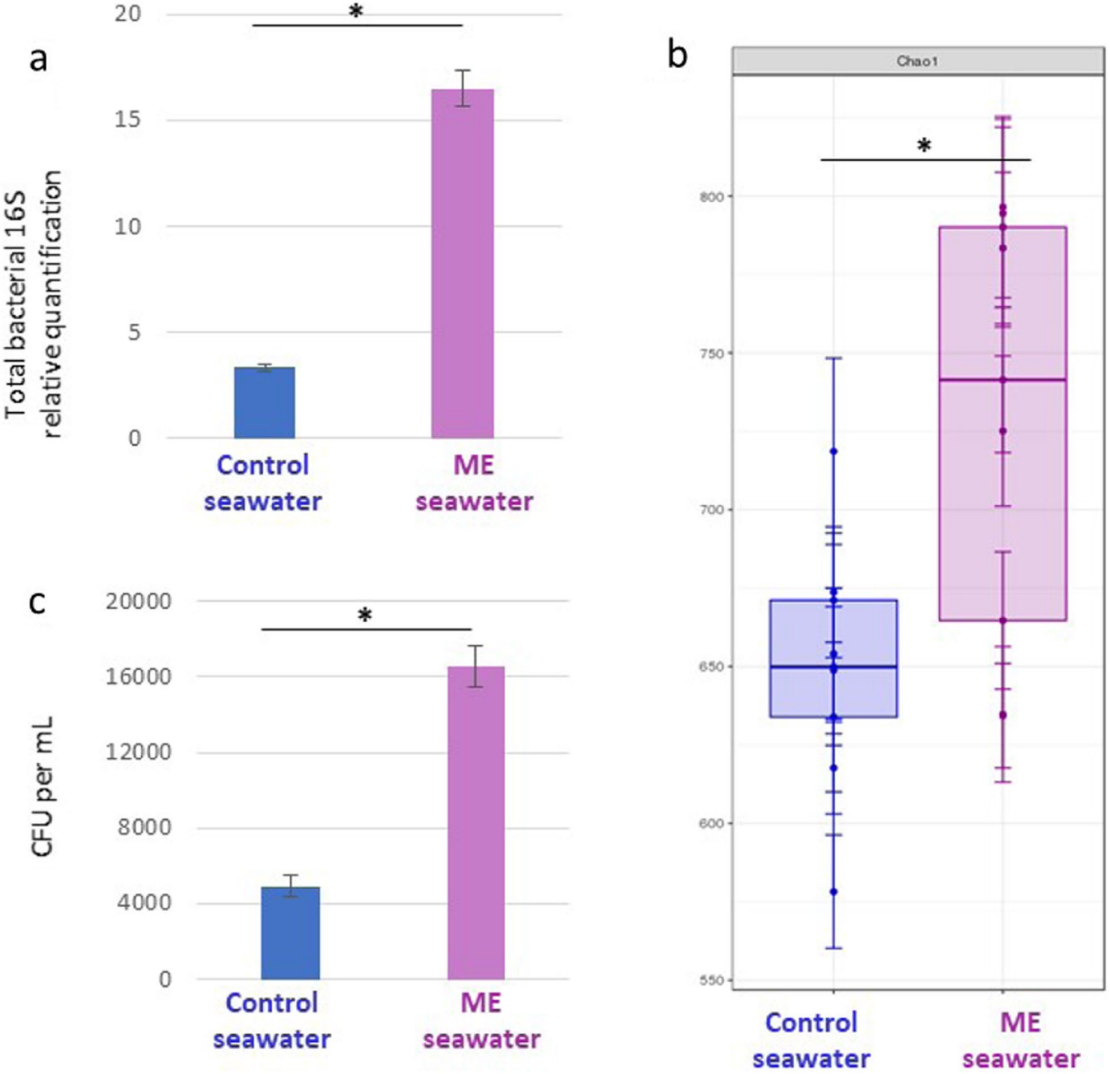
Donor oysters enriched microbial content of the seawater. **a** Bacterial 16S relative quantification (*y*-axis) of the control (blue) and ME seawater (purple) at day 2 of the exposure (*x*-axis). *Represents statistical significant differences between conditions: unpaired *t*-test with Welch’s correction, *p*-val = 0.001. **b** The Chao1 index (*y*-axis) from 16S barcoding analysis performed on the bacterial community of the control seawater (blue) and ME seawater (purple) sampled at day 2 of the exposure (*x*-axis). *Represents a statistically significant change in Chao1 index in the ME compared to the control seawater, i.e, ANOVA, *p*-val < 0.05. **c** Bar graph of cumulative CFU per mL (*y*-axis) of cultivable bacteria sampled in seawater until day 10 in the three tanks used in the control seawater (blue) and ME seawater (purple) (*x*-axis). *Represents a significant statistical difference in the ME compared to the control seawater, i.e., Wilcoxon test, *p*-val < 0.05

The bioinformatic pipeline for barcoding data treatment is represented in Fig. 2 of the Additional file 1.

Community analysis was performed on R software (R Core Team, 2013) using the phyloseq package [42]. Rarefaction curves of species richness were produced using the rarefy-even-depth and ggrare functions [42]. One-way ANOVA or nonparametric Kruskal-Wallis test (when the normality of residuals was rejected (Shapiro test)) was used to compare alpha diversity indices between conditions. When the ANOVA or Kruskal-Wallis tests were significant, we performed pairwise comparisons between group levels with the pairwise *t*-test or the Dunn test (post hoc analyses) using Bonferroni corrections for multiple testing. The significance threshold was set at 0.05 for all analyses. Principal coordinate analyses (PCoA) were computed to represent dissimilarities between samples using the Bray-Curtis distance matrix (beta diversity). Multivariate analysis of variance was tested using 999 permutations (adonis2 and betadisper from vegan package [43]). To compare the proportions for each genus between ME and control seawater or between ME-exposed and control oysters at day 2, we used the table of sum of sequences. We performed the analysis on the counts per sample of OTUs. Since we have three filter sizes for the water samples, we calculated the mean of the total read counts for the three filter sizes per genera. We used DESeq2 to identify the genera that exhibited a significant difference in their relative abundance in ME *vs*. control seawater or in ME-exposed *vs*. control oysters [44].

In order to identify bacterial taxa which were significantly overrepresented in the microbial community of the ME-exposed or control oysters sample, the “LDA Effect Size” (LEfSe) method [45] was used with a normalized relative abundance matrix. This method uses a Kruskal-Wallis followed by Wilcoxon tests (*p*-value ≤ 0.05) and then perform a linear discriminant analysis (LDA) and evaluate the effect size. The taxa with a LDA score greater than 2.0 were considered as biomarkers of exposure. A separate analysis was performed for larvae (pooled day 2 and day 10 samples) and juveniles (pooled samples for day 58 and day 120 H0) because of the strong developmental effect on bacterial composition. To increase the statistical power, samples of the F1 and F2 generation have been pooled.

### Transcriptome analysis (RNA-seq)

**Fig. 3:**
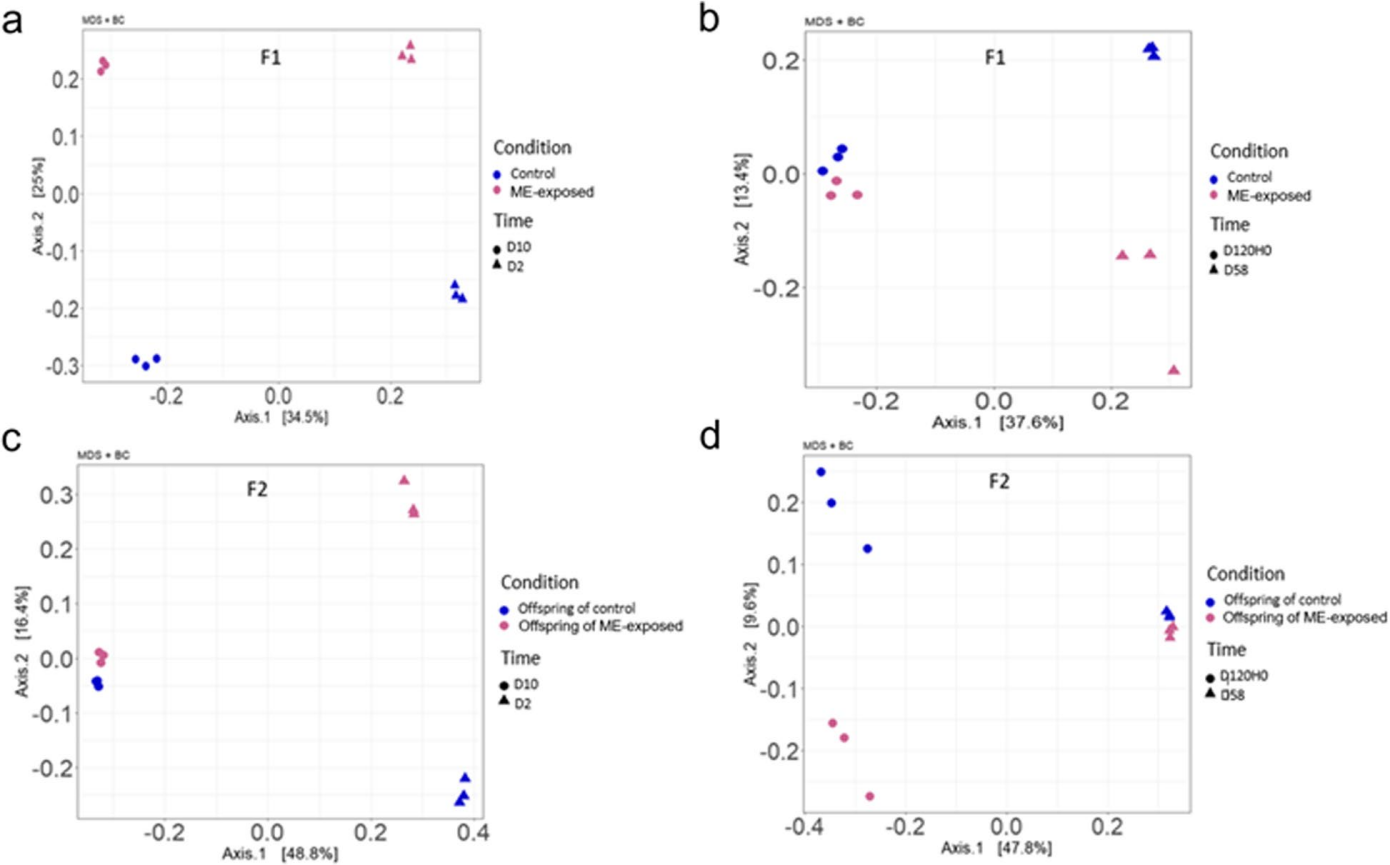
Long-lasting modification in *C. gigas* microbiota composition occurred following exposure to microorganism-enriched seawater. Principal coordinate analyses (PCoA) representing dissimilarities between samples using the Bray-Curtis distance matrix performed on 16S barcoding data obtained from oysters’ larvae (**a**, **c**) sampled at day 2 (D2, dots) and day 10 (D10, triangles) and from juvenile oysters (**b**, **d**) sampled at day 58 (D58, dots) and day 120 (D120H0, triangles) for generation F1 (**a**, **b**) and F2 (**c**, **d**). Differences between ME-exposed (pink) and control (blue) oysters were statistically significant for both larval (days 2 and 10) (*p*-val = 0.005 for F1; *p*-val = 0.001 for F2) and juvenile oysters (days 58 and 120) (*p*-val = 0.002; *p*-val = 0.005) from both generations

Samples used for RNA-Seq analysis are indicated in Table 2 of the Additional file 1. For each time point, 3 biological replicates were sequenced. RNA-Seq library construction and sequencing were performed at McGill University (Génome Québec Innovation Centre, Montréal, Canada) (http://www.genomequebec.com). NEB mRNA-stranded libraries were constructed and sequenced on a Hiseq 4000 (Illumina), in paired-end reads of 2 × 100 bp. The bioinformatic pipeline for RNA-seq data treatment is represented in Fig. 3 of the Additional file 1, and quality of the metrics is indicated in Additional file 2. Functional annotation and enrichment analysis were performed with RBGOA using an adaptive clustering and a rank-based statistical test (Mann-Whitney *U*-test) combined to the adaptive clustering [46]. “–log(qval)” (obtained from the Deseq2 analysis) was used as input for the RBGOA analysis to represent repressed or induced genes in ME-exposed compared to control oysters. The R and Perl scripts used can be downloaded at https://github.com/z0on/GO_MWU [47]. Significantly, enriched biological processes were expressed as a ratio between the number of genes differentially expressed divided by the total number of genes assigned to that biological process and was represented in heatmaps with MeV [48].

Because not all known *C. gigas* antimicrobial peptides (AMPs) were present in the *C. gigas* reference genome (assembly version V.9), read counts for all of the time points were specifically obtained by alignment against a protein database which contains the AMP sequences using DIAMOND 0.7.9 [49], and a differential analysis between ME-exposed vs. control oysters was performed as previously described [28].

### Genetic analysis

**Fig. 4:**
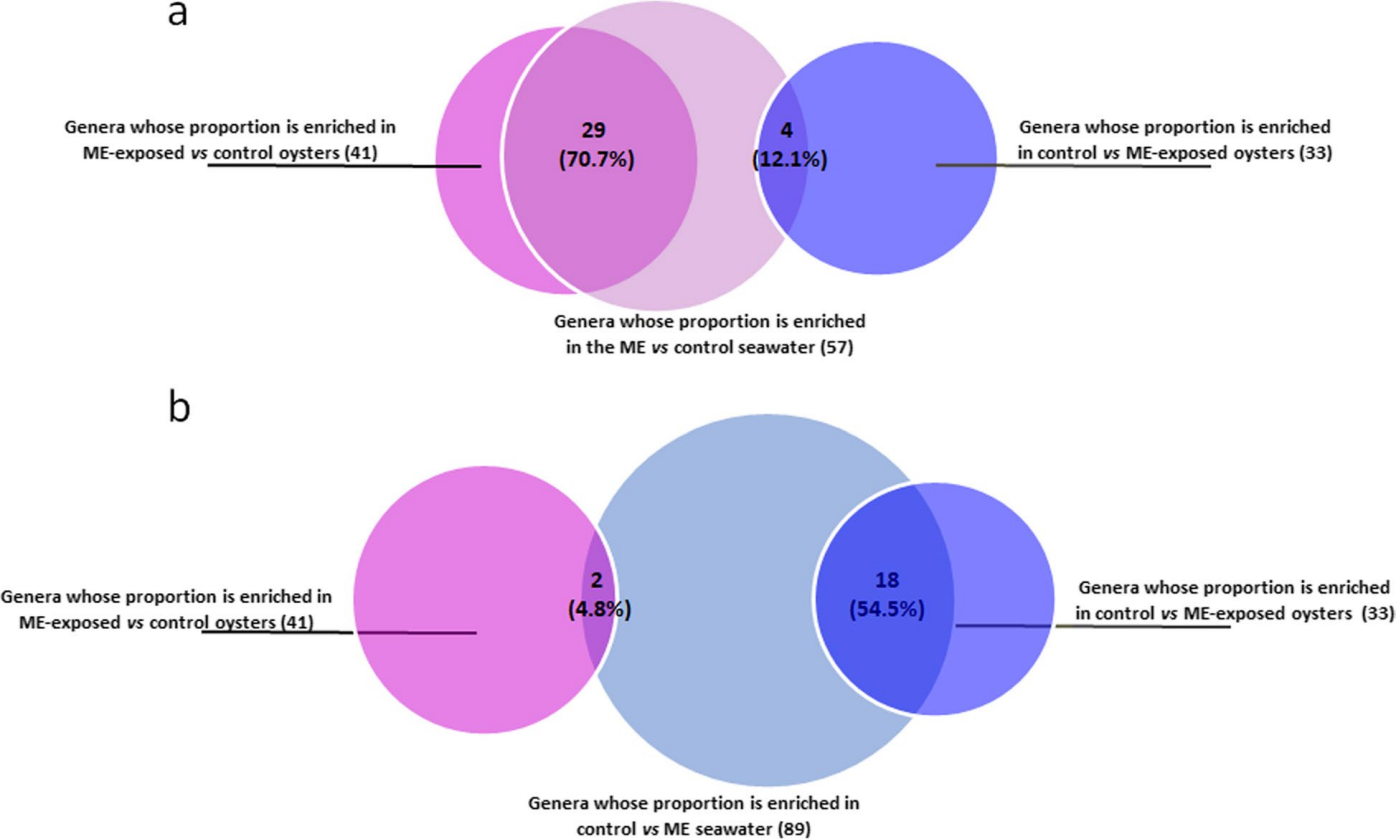
The bacterial taxonomic profile of microorganism-enriched seawater and recipient oysters indicates microbial colonization from seawater. **a** Venn diagram indicating the number of genera which displayed a higher representation in ME vs. control seawater (centered light pink circle, *n* = 57) which are common to the genera which displayed a higher representation in the ME-exposed compared to control oysters (left pink circle, *n* = 41) or *vice versa* (right blue circle, *n* = 33). **b** Venn diagram indicating the number of genera which displayed a higher representation in control vs. ME seawater (centered light blue circle, *n* = 89) which are common to the genera which displayed a higher representation in the ME-exposed compared to control oysters (left pink circle, *n* = 41) or *vice versa* (right blue circle, *n* = 33). Comparison of the total read count for each bacterial genus in ME-exposed vs. control oysters and in ME vs. control seawater was performed on samples of day 2 of the F1 generation. Statistically significant differences were identified with DESeq2

gDNA shotgun library construction and Hi-seq sequencing (Illumina, paired-end reads of 150 bp) were done at McGill University (Génome Québec Innovation Centre). Bioinformatic pipelines used for genetic analysis are described in Fig. 4 of the Additional file 1. A pool of 30 oysters was used to generate the genetic data. Quality metrics are indicated in Additional file 2. Principal component analyses (PCA) were generated with R software (R Core Team, 2013) from the allele frequency matrix using R packages “dplyr” [50], “tidyr” [51], “ggplot2” [52], “RcolorBrewer” [53], and “mixOmics” [54, 55]. Evidence for adaptive selection at each SNP was tested using the FLK statistic [56], using a modification of the hapFLK software [57] allowing to input allele frequencies instead of individual genotypes [58]. The FLK statistics were computed based on the comparison of allele frequencies in the exposed and control oysters. This analysis was performed independently for the two generations, F1 and F2. Distributions of FLK *p*-values were plotted with R. Significant SNPs were called at a false discovery rate (FDR) of 5%, 10%, 15%, and 20% following the approach of a previous study [59], implemented in the *q*-value R package.

### DNA methylation analysis

**Fig. 5:**
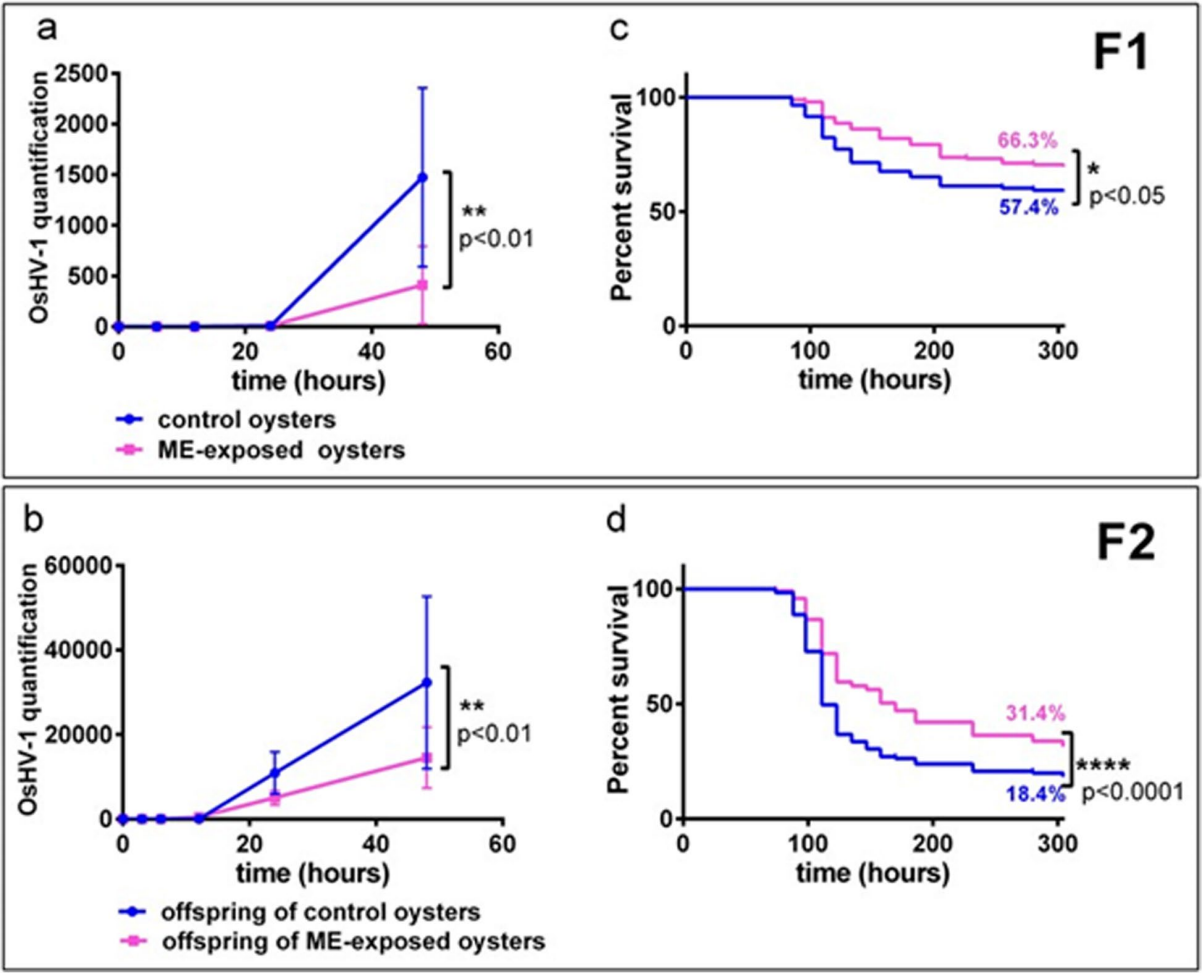
Oysters exposed to microorganism-enriched seawater and their offspring displayed enhanced survival when challenged with POMS. **a** and **b** Quantification of OsHV-1 DNA (*y*-axis) during experimental infection (in hours = *x*-axis) in ME-exposed (pink line) and control (blue line) oysters for generation F1 (**a**) and generation F2 (**b**). *p*-value of two-way ANOVA with Bonferroni’s multiple comparisons test is indicated. **c** and **d** Survival curves of oysters (*y*-axis) during experimental infection (in hours = *x*-axis) in ME-exposed (pink line) and control (blue line) oysters for generation F1 (**c**) and generation F2 (**d**). *p*-val of log-rank test and final survival percentage for each condition are indicated on each survival curve

Bisulfite conversion, BS-seq paired-end library construction, and sequencing were performed at McGill University (Génome Québec Innovation Centre). Sequencing was performed on a HiseqX using 150 nucleotide paired-end reads. Quality metrics are indicated in Additional file 2. The bioinformatics pipeline for BS-seq analysis is represented in Fig. 5 of the Additional file 1 and was performed on the local Galaxy platform [60] (http://bioinfo.univ-perp.fr). Differential methylation analyses were performed with DMRseq package [61]. Since this software is generally applied to vertebrate DNA methylation, the parameters were optimized using the DMRsim package in order to optimize the detection of true positives in our dataset. The DMRsim package was used to simulate differential methylation analysis on 180 DMRs artificially generated out of a dataset containing 700,000 methylated CpG using a cutoff value of 0.01. The best parameters (blocksize = TRUE, minnumregion = 3, deltamax = 0.25, bpspan = 1000, mininspan = 10, maxgapssmooth = 2500, smooth = TRUE) allowed for detection of 50% of true positives with 0% of false positives for a *p*-val < 0.05 in our dataset and were used for the differential methylation analysis. The R scripts used here can be downloaded at https://github.com/IHPE/DMRseq_wrapper. Statistically significant differentially methylated regions (DMRs) were checked by visual inspection using the Integrative Genomics Viewer (https://software.broadinstitute.org/software/igv/). DMRs genomic positions were intersected with the annotation of *C. gigas* genome version 9 [62] to identify DMRs that occurred within genes (differentially methylated genes (DMGs)) and within promoters (differentially methylated promoters (DMPs)). The +2 kb region upstream of the transcription start site was defined as the promoter position. DMGs were used for functional annotation and enrichment analysis with RBGOA. A binary analysis was applied: a 1 score and a 0 score were attributed to each statistically significant or not significant DMG respectively, whatever the sense of the change in methylation level. The R and Perl scripts used here can be downloaded at https://github.com/z0on/GO_MWU [47]. The following parameters were used for the adaptive clustering: largest = 0.2; smallest = 5; clusterCutHeight = 0.25. Statistically significantly enriched biological processes were classified manually into larger biological functions. Biological processes were graphically represented using Multiple Experiment Viewer (MeV). The color intensity represents the ratio: number of genes differentially methylated divided by the total number of genes assigned to that biological process.

Differential methylation for genes related to immune functions belonging to IFN signaling pathway, JAK-STAT pathway, nucleic acid recognition, and RNAi pathway was graphically represented with MeV. The color intensity represents the *p*-val obtained with the DMRSeq analysis.

The number of heritable DMRs was determined using bedtools intersect -a F2_DMRs.bed -b F1_DMRs.bed -wo | wc −l. To test whether DMRs are inherited in a statistically significant manner, 5000 BED files with regions of identical size and number of DMRs as for the F1 generation were generated and intersected with the F2 real DMRs. Five-thousand bootstrapping tests of heritability were performed by 5000 iterations of bedtools shuffle -g cg9.len -i F1_DMRs.bed -maxTries 1000 that were then used as the -b file in bedtools intersect. Mean value and standard deviation were calculated for these 5000 intersections and were compared to the value that was obtained from the real dataset. Standard deviation for the real dataset was assumed to be the same percentage as the one obtained on the shuffled data. Based on this mean value and standard deviation, a *t*-test testing the null hypothesis was performed, and the null hypothesis was rejected if the absolute value of the statistical test was greater than 3.090, a critical value expected for a sample size above 100 (https://www.itl.nist.gov/div898/handbook/eda/section3/eda3672.htm).

## Results

Exposing oyster larvae to microorganism-enriched seawater shifts their bacterial microbiota throughout their life span and in the next generation.

To investigate whether the rich surrounding microbial environment that oyster larvae face early in life could influence the trajectory of the oyster immune response, we developed an experimental setup to compare the effects of control or microorganism-enriched seawater environments during early larval development. Pathogen-free larvae (F1 generation) were produced in a bio-secured (filtered and UV-treated seawater) hatchery. A subset of F1 larvae were exposed from 2 h to 10 days post-fertilization to a microorganism-enriched environment by cohabitation with oysters transferred from a natural environment during a POMS-free period (microorganism-enriched seawater, ME seawater, Fig. 1). As a control, a subset of F1 larvae were raised in bio-secured conditions with no cohabitation or no microbial exposure (control seawater, Fig. 1). From 10 days onward, both ME-exposed and control oysters were raised in the same bio-secured conditions. A part of these two oyster subsets were maintained in bio-secured conditions and reproduced, 1 year later, to generate the F2 generation (Fig. 1). Between the ME and control oysters, we observed equivalent developmental success and survival rate in the F1 generation (Table 1 of the Additional file 3). Moreover, the absence of OsHV-1 was confirmed for ME and control oysters during exposure time. The nature of the seawater treatments was evaluated by analyzing the bacterial load and composition of the ME and control seawater by qPCR targeting 16S rRNA genes and 16S barcoding at day 2 post-fertilization. As expected, the ME contained fivefold more total bacteria than the control seawater (unpaired *t*-test with Welch’s correction, *p* = 0.001) (Fig. 2a) and carried a more diverse microbiota as evidenced by the Chao1 index (ANOVA, *p* < 0.05) (Fig. 2b). This trend was confirmed by plating the seawater sampled in each tank containing the recipient larvae on marine agar, revealing that ME tanks contained 3.4 times more cultivable bacteria (16544 CFU/ml) than the control seawater tanks (4916 CFU/ml) (Wilcoxon test: *p* < 0.05) (Fig. 2c). This bacterial content in the breeding pipe reflects what is met in natural seawater [63]. Altogether, the ME condition was considered as an exposure to a safe, microorganism-enriched environment that mimics the natural seawater.

To test the immediate and long-term impact of early ME exposure on the oyster microbiota, we analyzed the bacterial community composition by 16S amplicon sequencing in both F1 and F2 whole body oysters (Additional file 4). Differences in composition and diversity were evidenced between ME-exposed and control oyster larvae during the ME seawater exposure (Fig. 3a, Fig. 1 of the Additional file 3). Among the 41 genera that had higher relative abundance in the ME-exposed larvae compared to control larvae, 29 (70%) were also more highly represented in the ME seawater (Fig. 4, Additional file 5). Conversely, among the 33 genera that make up a higher proportion in the control compared to ME-exposed oysters, 18 (54.5%) were also more represented in the control seawater, strongly suggesting that the microorganisms from the ME seawater colonized the oyster larvae during the exposure.

Dissimilarity analysis, based on the Bray-Curtis index, showed that the oyster microbiota profiles clustered first by developmental stage (Fig. 2 of the Additional file 3) and then by treatment (Fig. 3a to d). This analysis indicated that the microbiota composition of ME-exposed vs. control oysters was significantly different, not only during the exposure (Fig. 3a, permutation test *p*-val = 0.002) but also several months later during the F1 juvenile stage (Fig. 3b permutation test *p*-val = 0.005) as well as during larval and juvenile stages of the F2 generation (Fig. 3c and d, permutation test *p*-val = 0.001 and 0.005, respectively). We further performed a differential analysis based on OTU relative abundance in order to identify bacteria which would be overrepresented in the microbiota of the ME-exposed or control oysters in both generations. We found 31 and 16 taxa showing statistically significant overrepresentation in the ME-exposed and control oysters respectively at larval stages and 8 and 7 taxa showing statistically significant overrepresentation in the ME-exposed or control oysters respectively at juvenile stages (Fig. 3 of Additional file 3 and Additional file 4). Gender detected in larvae which are associated with increased resistance in both generations are as follows: *Marinibacterium*, *Halodesulfovibrio*, *Cyclobacteriaceae*, *Marinobacterium*, *Psychroserpens*, *Pelagibaca*, *Ekhidna*, *Kordia*, *Crocinitomix*, *Lacimonas*, *Changchengzhania*, *Massilia*, *Olleya*, *Leisingera*, *Vitellibacter*, *Octadecabacter*, and *Shewanella*. Gender detected in juveniles which are associated with increased resistance in both generations are as follows: *Neptunomonas*, *Cobetia*, and *Sphingoaurantiacus*. None of the OTU enriched in ME-exposed larvae is found also enriched in ME-exposed juveniles, which underline that there is a microbiota signature specific in each stage that may be responsible for increased survival.

Taken together, this barcoding analysis clearly indicated that the oyster microbiota significantly shifts across developmental stages, but despite this strong developmental effect, the ME seawater exposure during larval stages induced a persistent modification of the oysters’ bacterial microbiota composition that even persisted in the subsequent generation.

### Early life microbial exposure primes intergenerational immunity against Pacific oyster mortality syndrome

To test whether ME exposure of oyster larvae can produce a long-term impact on their resistance to disease, we conducted an ecologically realistic experimental infection mimicking the Pacific oyster mortality syndrome (POMS) on juvenile oysters from F1 and F2 generations (Fig. 1 of the Additional file 1) [28, 36]. The number of surviving oysters was monitored for 300 h, while oyster OsHV-1 load was measured before the onset of the mortalities (Fig. 5). The increase in virus load during the first 48 h confirmed successful infection. The viral load was significantly lower in the ME-exposed oysters or their offspring compared to the control lineage (*p*-val of two-way ANOVA with Bonferroni’s correction for multiple comparisons test: *p*-val < 0.01) (Fig. 5a and b). Consistent with these results, we observed that ME-exposed oysters had a better survival rate compared to controls in both F1 (66.3% vs. 57.4%, log-rank test, *p*-val < 0.05) and F2 generations (31.4% vs. 18.4%, log-rank test, *p*-val < 0.0001) (Fig. 5c and d, respectively). These results were confirmed in a parallel field infection test conducted with oysters from both F1 and F2 generations (F1: 16.4% vs. 14%, log-rank test, *p*-val < 0.001, F2: 8.5% vs. 1%, log-rank test, *p*-val < 0.0001) (Fig. 4 of Additional file 3).

### No evidence for genetic selection as the mechanism of increased immune capacity

**Fig. 6:**
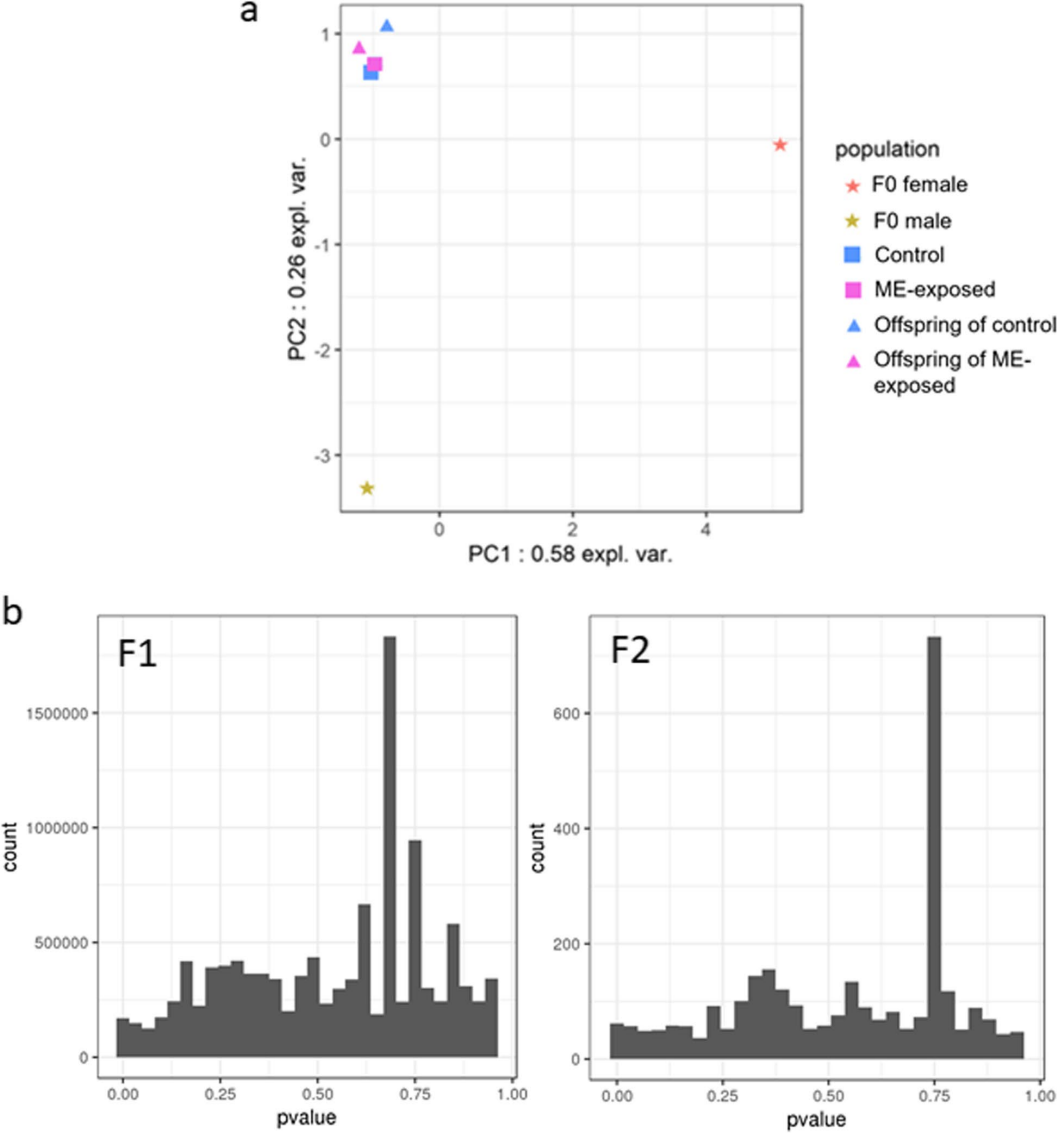
Larval exposure to microorganism-enriched seawater did not result in genetic selection. **a** PCA of genome-wide SNP allele frequencies in F0 female (orange) and male (yellow) gametes (star) leading to generation F1 and from F1 (square) and F2 (triangles) generations of the control (blue) and ME-exposed (pink) lineages. **b** Histogram of *p-*val obtained when comparing allele frequencies between the ME-exposed and control oysters using the FLK test, for generations F1 (left) and F2 (right). *P-*val was plotted using R. Each sample included 30 pooled juvenile individuals sampled at day 120

We investigated if a genetic selection could have occurred through ME exposure and would have selected more resistant oysters based on specific allele associations. To this end, we evaluated genome-wide SNP allele frequencies in juvenile oyster samples using whole genome sequencing (WGS). Principal component analysis (PCA) of these data showed little genetic divergence between the ME-exposed vs. control oysters for the F1 and F2 generations (Fig. 6a). Next, we conducted a genome scan comparing allele frequencies in ME-exposed vs. control oysters using the FLK test to interrogate any signals of positive selection. The FLK statistic considers genome-wide allele frequency data in a set of populations and aims at detecting positions where genetic differentiation between these populations is higher than expected under neutral evolution. It returns for each SNP a *p*-value allowing to reject or accept neutrality. In the case of genetic selection on some SNPs, an excess of low *p*-values is expected. No such excess was detected here, revealing an absence of genetic selection between exposed and control lines for the F1 and F2 generations (Fig. 6b). Furthermore, no significant SNPs could be detected based on a FDR value below 0.05 (even below 0.15 for F2, Table 2 of the Additional file 3). This absence of genetic selection is consistent with the fact that the survival rate of ME-exposed larvae was not significantly lower than of control larvae (Table 1 of the Additional file 3). Altogether, these findings indicate that genetic alterations are not responsible for the increased resistance among the ME-exposed oyster lineage.

### Upregulation of immune-related and other transcripts in microbially exposed oyster lineages

Next, we asked whether ME exposure impacted oyster gene expression by performing transcriptomic analyses on larvae and on juveniles just before and during the POMS disease breakouts for both F1 and F2 oysters. During ME exposure in the F1 larval stages, we observed a large shift in gene expression (3410 and 1100 DEGs at days 2 and 10) (Table 3 of the Additional file 3 and Additional file 6). However, the difference in gene expression between ME-exposed and control oysters is much more nuanced at juvenile stages (35 DEGs at day 120). These observations were similar to what we observed in the F2 generation (6029 DEGs at day 10 and 120 DEGs at day 120).

**Fig. 7:**
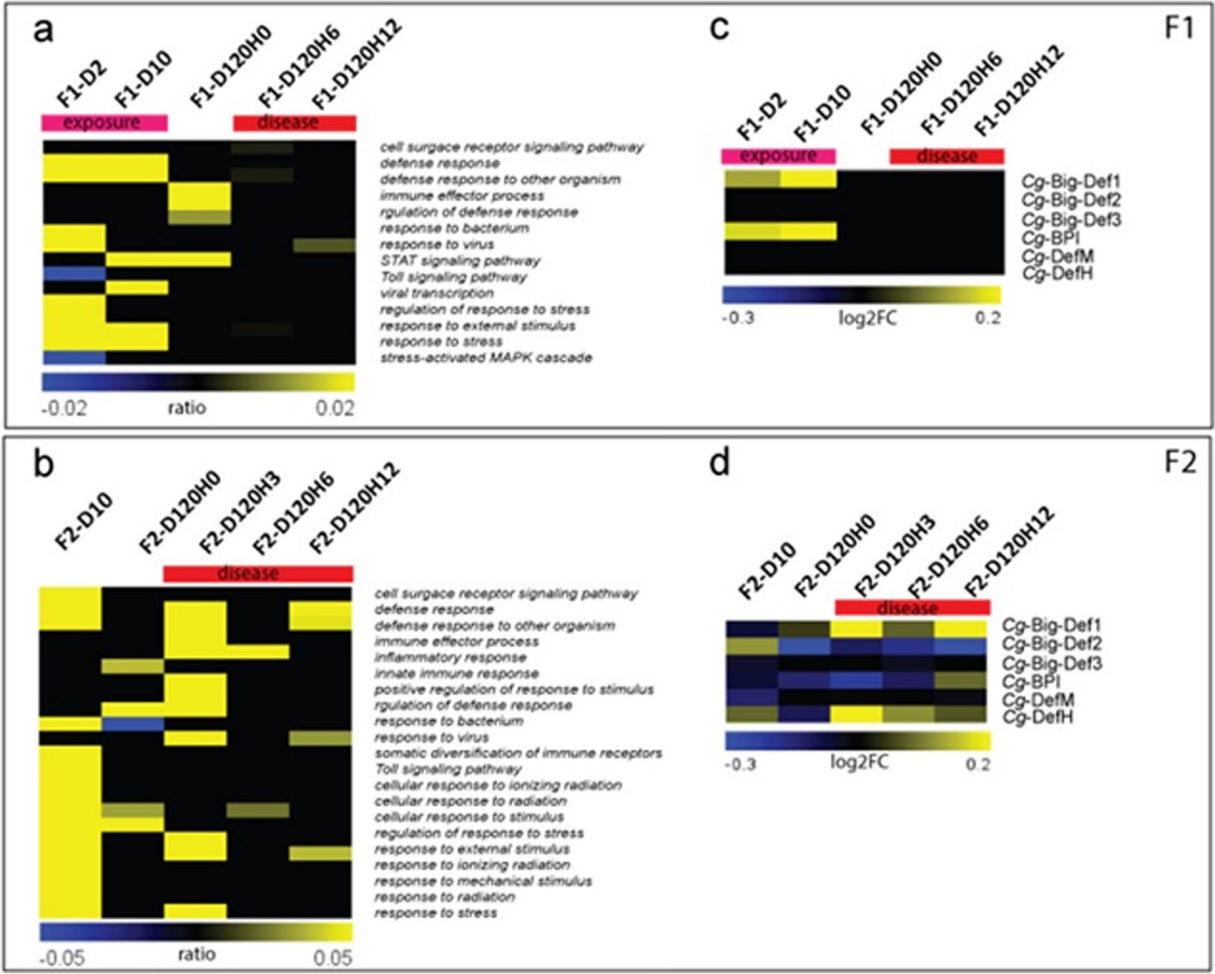
Long-lasting and intergenerational changes in immune gene expression resulting from early life microbial exposure. Significantly differentially expressed genes in ME-exposed compared to control oysters were obtained after DESeq2 comparison. Functional annotation with RGBOA performed on the DEG led to the identification of biological functions which were statistically significantly enriched in the exposed compared to control samples. **a** and **b** The heatmap represents the immune-related functions enriched in F1 (**a**) and F2 generation (**b**). The heatmap depicts the ratio of the number of significant DEGs within a biological function divided by the total number of genes belonging to that biological function for each sample analyzed. Positive ratios indicate an over-representation of a function (yellow on the heatmap), and negative ratios indicate an under-represention of a function (blue on the heatmap). **c** and **d** A special focus on genes encoding antimicrobial peptides that are differentially expressed between ME-exposed and control oysters (**c**) and between offspring of ME-exposed and offspring of control oysters for generation F2 (**d**). The heatmap depicts the log2FC of significant DEGs obtained according to the diamond bioinformatic pipeline. Overexpressed and underexpressed AMP-related genes are represented by yellow or blue color, respectively. In the F1 generation, analysis have been performed on oysters sampled during the exposure at day 2 (F1-D2) and day 10 (F1-D10), at juvenile stage just before the disease induction (F1-D120H0) and during the disease process after 6 and 12h of disease induction (F1-D120H6, F1-D120H12). In the F2 generation, analysis has been performed in larvae at day 10 (F2-D10), just before the disease induction (F2-D120H0) and during the disease process after 3, 6, and 12h of disease induction (F2-D120H3, F2-D120H6, F2-D120H12)

To investigate which biological processes are modulated by ME exposure, we performed a rank-based gene ontology analysis (RBGOA; false-discovery rate [FDR] < 0.01) (Additional file 7). The broad gene expression shift in larval oysters encompassed many functional annotations, including general cellular process, metabolism, response to environmental stimulus, infection and immune response, transcription and gene expression, development, cell fate, RNA process, translation and protein processing, signal transduction, and transport. In juvenile oysters of both generations, upregulation of genes involved in responses to external stimuli and immunity persisted from the larval stage, suggesting a potential role for these genes in mediating resistance to POMS at the time of infection. During POMS disease onset at the juvenile life stage, we observed a strong overrepresentation of immune functions, especially in the F2 generation (Fig. 7b). Analysis of the individual genes driving this enrichment revealed gene families typically involved in microbial-associated molecular pattern (MAMP), recognition (PGRP, lectins, scavenger receptors, TLR, RLR, macrophage receptor), innate immune pathways (components of IFN-TLR-JAK/STAT pathways as MyD88, IRF2, STING), interaction with bacteria (dual oxidase), and antimicrobial effectors (TNF, proteinases, SOD, interferon-stimulated genes) (Additional file 6). These immunity-linked families were found differentially expressed in both F1 and F2 generations, especially at larval stages, meaning that the offspring of ME-exposed oysters has inherited the capacity for an improved immune gene expression, although these oysters have not been exposed *per se*. Importantly, the individual genes encoding for these immune functions were generally different in F1 compared to F2 generation (different CGI numbers). In addition, a closer look at antimicrobial peptides or proteins (AMP) expression revealed a significant overexpression in ME-exposed compared to control oysters, either during the exposure period at larval stages in F1 (Big-Def1 and BPI at day 2 and day 10) (Fig. 7c) or in F2 (Big-Def2 and DefH at day 10) (Fig. 7d).

Apart from immune functions, our transcriptomic analysis highlighted that ME exposure during larval stages also affected key metabolic pathways. The expression of genes encoding for enzymes involved in glycolysis and the TCA cycle was lower in both generations during the larval stages, whereas the oxidative phosphorylation pathway and folate metabolism enzymes were downregulated at day 10 of the F2 generation only (Additional file 8). This shift in metabolism is specifically observed in larval stages since ME-exposed and control juveniles display the same metabolic gene expression pattern. We also observed that functions linked to chromatin structure (RBGOA analysis, Additional file 7) were repressed at day 10 of the F2 generation and genes encoding for folate metabolism, and DNA methylation machinery enzymes were repressed at day 120 of the F2 generation (Additional file 8).

Taken together, these transcriptomic analyses showed that the ME seawater larval exposure of *C. gigas* resulted in modification of the immune response of the oysters. This immunomodulatory effect was maintained up to the juvenile stages and in the subsequent generation. These results support the idea that transcriptional changes may be responsible for the increased immune capacity that we observed in the survival assay.

### Differences in DNA methylation may explain the transcriptional changes observed in microbially exposed oyster lineages

The observed multigenerational impact of the ME exposure on oyster survival capacities and transcriptomic response, as well as the absence of genetic selection between conditions, led us to investigate the impact on epigenetic information through analysis of differentially methylated regions between ME and control oyster lineages. Whole genome bisulfite sequencing (WGBS) analysis was performed on oysters sampled at days 10 and 120 of the F1 and F2 generations (Table 2 of the Additional file 1). We found that *C. gigas* DNA was mainly methylated in a CpG context with a mosaic-type cytosine methylation pattern as previously described [64]. The PCA results of the global pattern of cytosine methylation data showed a clustering according to developmental stages but not according to the treatment (ME-exposed or control oysters) (Fig. 5 of the Additional file 3). This result suggested that the cytosine methylation pattern changed during development as previously observed by others [65], and this observation was confirmed by a global decrease in the cytosine methylation level observed from larval to juvenile stages (1.77 to 1.58% for F1 and 1.82 to 1.56% for F2, respectively, *p*-val from Wilcox test < 0.01 for both generations) (Fig. 6 of the Additional file 3). Although ME exposure did not appear to strongly affect the level of cytosine methylation at the genome wide scale (Fig. 6 of the Additional file 3), a trend toward a hyper-methylation was observed in ME-exposed compared to control larvae in the F1 generation. In contrast, an opposite trend toward a hypo-methylation was observed in F1 juveniles and in F2 larvae (Fig. 6 of the Additional file 3).

To gain deeper insights into the impact of the ME exposure on methylation patterns of oysters, we used DMR-Seq software [61] to identify differentially methylated regions (DMRs) between ME-exposed and control oysters for each generation. The differential methylation analysis led to the detection of 4325 and 5531 DMRs for larvae (day 10) of the F1 and F2 generation, respectively, and 4985 and 5207 for juveniles (day 120) of the F1 and F2 generation, respectively (Table 4 of the Additional file 3, Additional file 9). Hyper-methylated DMRs in ME-exposed compared to control oysters were more frequent than hypomethylated DMRs at day 10 of the F1 generation (57.4% hyper-methylated vs. 42.6% hypo-methylated DMRs). However, hypo-methylated DMRs in ME-exposed oysters were more frequent at day 120 of F1 generation (40.9% hyper-methylated vs. 59.1% hypo-methylated DMRs) and at day 10 of the F2 generation (22.2% hyper-methylated vs. 77.8% hypo-methylated DMRs) (Table 4 of the Additional file 3). These observations agreed with the previous trend observed at the genome-wide level (Fig. 6 of the Additional file 3).

**Fig. 8:**
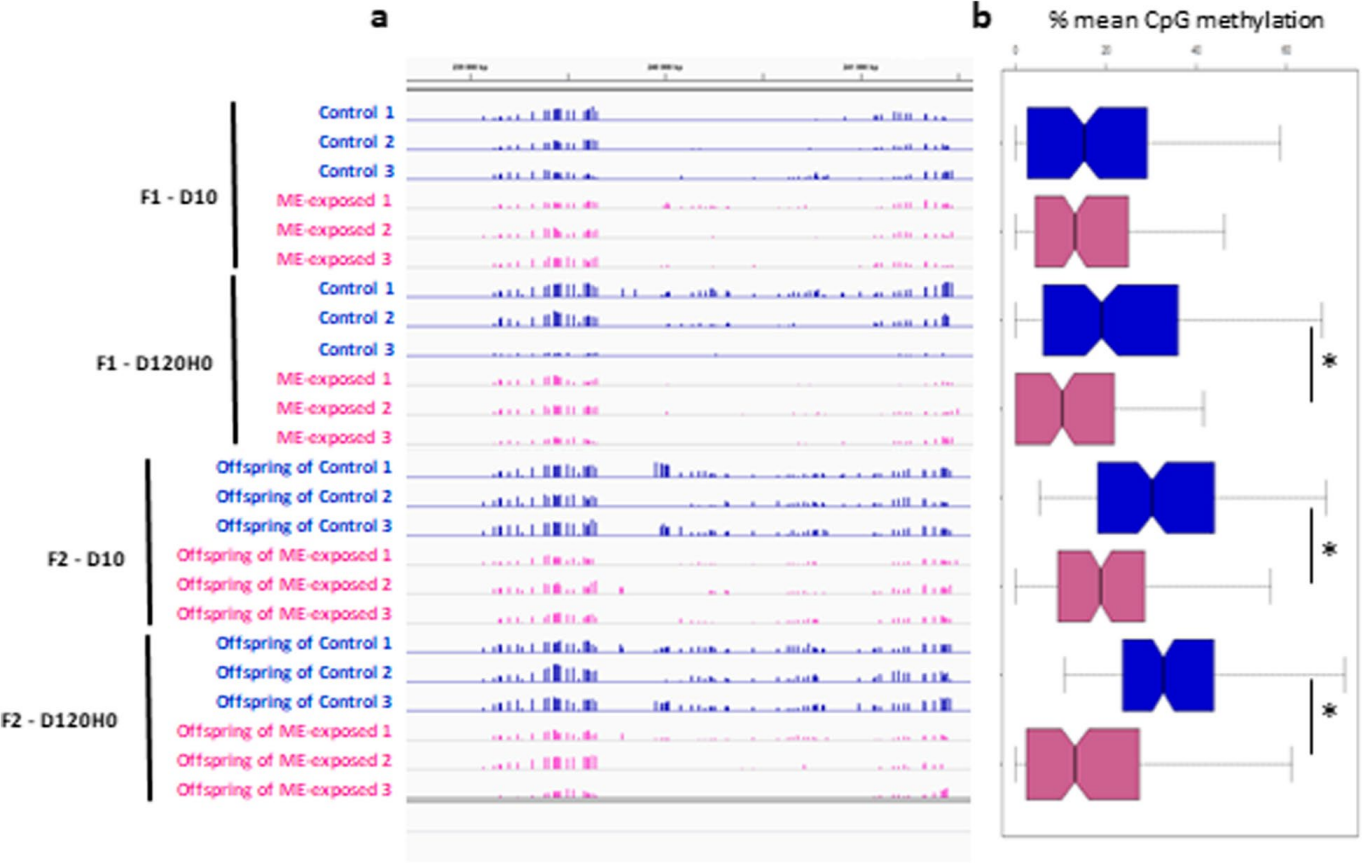
Local DNA methylation changes persist from F1 to F2 generation. **a** Snapshot of Integrative Genomics Viewer Windows which displays CpG methylation data from scaffold 1792:239,000–242,000 for oysters sampled at day 10 of F1 and F2 generation (F1-D10, F2-D10, respectively) and at day 120 just before disease induction of F1 and F2 generation (F1-D120H0, F2-D120H0, respectively). Each bar indicates the level of methylation at a CpG position (*x*-axis) on a scale from 0 to 100% (*y*-axis). The three biological replicates for each time point are represented. The hypomethylation displayed in the F1 juvenile oysters (F1-D120H0) in ME exposed (pink color) compared to control oysters (blue color) was detected with the DMRseq software and was inherited from F1 to F2 generation in larvae (F2-D10) and juveniles (F2-D120H0). **b** Box plots representing the distribution of the level of methylation (mean of three biological replicates) for each CpG position present in the selected region. *Indicates a *p*-val < 0.05 of a Mann-Whitney analysis comparing the ME exposed and control oysters for time point

Next, we analyzed DMRs that intersected with gene positions, defining these regions as differentially methylated genes (DMGs), and asked which functional annotations are overrepresented among DMGs. According to this analysis, the functions mostly impacted by DNA methylation changes were related to general cellular process, metabolism, response to environmental stimulus, signal transduction, translation, and protein processing and development (Additional file 10). Similar functions were found to be modified in the oyster transcriptome in response to the ME exposure (Additional files 7 and 10). Although immune functions were not statistically highlighted by the RBGOA analysis, 128 DMGs were found in genes encoding for immune functions. Genes coding for the interferon pathway, immune signaling pathway, viral production, and ubiquitin modification displayed changes in their cytosine methylation profiles (Fig. 7 of the Additional file 3). However, we did not observe a canonical association between expression levels and methylation changes when analyzing correlations between methylation and transcriptome profiles (Fig. 8 of the Additional file 3).

Some DMRs were meiotically inherited from the F1 to the F2 generations (Fig. 8). Forty-eight hyper-methylated DMRs and 120 hypo-methylated DMRs were conserved from F1 to F2 at the larval stage, and in the juvenile stage, we detected 147 hyper-methylated DMRs and 252 hypo-methylated DMRs conserved from F1 to F2 (Table 1). To test whether this number of meiotically heritable DMRs was higher than would be expected by chance, we randomly generated 5000 files containing artificial DMRs of identical size and number as the DMRs detected in the real dataset of the F1 generation and intersected these with the real dataset of the F2 DMRs. Mean values of the number of intersections between F2 DMRs and these randomized regions were always significantly lower than the number of intersections between F1 and F2 DMRs from the real dataset (Table 1 and Additional file 11), indicating that the similarity of DMRs in F1 and F2 did not occur by chance.

**Table 1:** Shared DMRs between F1 and F2 generations are likely the result of intergenerational epigenetic inheritance

**Day 10 hyper-methylation**		
	F1 ME-induced DMRs (*n* = 2482)	F1 randomly generated DMRs (*n* = 2482)
F2 ME-induced DMRs (*n* = 1230)	**48.0 ± 17.9**	**7.20 ± 2.7**
**Day 10 hypo-methylation**		
	F1 ME-induced DMRs (*n* = 1843)	F1 randomly generated DMRs (*n* = 1843)
F2 ME-induced hypo-DMRs (*n* = 4301)	**120.0 ± 27.8**	**18.2 ± 4.2**
**Day 120 hyper-methylation**		
	F1 ME-induced DMRs (*n* = 2040)	F1 randomly generated DMRs (*n* = 2040)
F2 ME-induced DMRs (*n* = 2550)	**147.0 ± 38.5**	**14.8 ± 3.9**
**Day 120 hypo-methylation**		
	F1 ME-induced DMRs (*n* = 2945)	F1 randomly generated DMRs (*n* = 2945)
F2 ME-induced DMRs (*n* = 2657)	**252.0 ± 48.1**	**27.6 ± 5.3**

Taken together, our epigenetic analysis shows that microbial exposure during larval stages impacts the DNA methylation pattern in both the directly exposed oysters as well as their offspring. The DNA methylation profile of genes involved in immune functions was clearly impacted in both generations, although a functional consequence on their expression was not evidenced. We showed that inheritance in the DMRs between F1 and F2 generation was not obtained by chance which suggests that DNA methylation changes can be inherited via epigenetic memory from the F1 to F2 generation.

The number of genomic coordinate intersections between either (a) F1 and F2 DMRs induced after the microbial exposure (ME-induced DMRs) or (b) mock randomly generated F1 DMRs (randomly generated DMRs) and F2 DMRs (ME induced DMRs) was compared by a *T*-test. Hyper- and hypo-methylated DMRs were tested separately within each developmental stage. See also Additional file 11.

## Discussion

A growing body of evidence shows that environmental pressure can be responsible for heritable phenotypic outcomes and changes in life history traits of living species [66]. Early life stages are considered a key window of opportunity during which individual experience with the surrounding environment can be integrated to change the phenotype at the intra- and trans-generational level [5, 8, 11, 33, 35, 67]. Proper establishment of the microbiota during this sensitive window plays a pivotal role for critical functions throughout the organism’s life span, such as the immune system [5, 8, 11, 68]. In the present study, we investigated the effect of a natural non-pathogenic microbial exposure during early larval development on the immune capacities of *C. gigas* in later life stages and in the next generation. We showed that oysters exposed to seawater enriched for microorganisms had a significantly greater capacity to prevent viral proliferation and to survive when exposed to the POMS disease later in life. This improved capacity was also observed in the offspring of these oysters, which themselves had not encountered any microbial exposure. We found that exposing larvae to ME seawater clearly modulated the overall oyster transcriptome, not only during the exposure but also after the exposure 120 days and even in the subsequent generation. Noteworthy, we identified strong and consistent differences in microbiota composition during development confirming community selection in the course of immune system maturation. This long-lasting effect supports the idea that transient microbial exposure during early larval development can positively influence the microbiota community and the immunity far beyond the exposure period. Recently, many examples of cross talk between the commensal microbiota and the host immune system have been reported [9, 10, 69–71], and increasing insights into underlying mechanisms have been obtained in invertebrate species which have an innate immune system only [72–75]. The systemic nature of the microbiota effect has been reported in several studies [76, 77]. In different vertebrate species, early exposure to commensal microbiota was found to increase their immunocompetence [9, 10, 69, 70] and to activate conserved immune pathways involved in both antibacterial and antiviral response (notably the Toll-NF-kB, JAK/STAT, and IFN pathway). These pathways have already been shown to be implicated in efficient immune response in oysters, and we show here that ME-exposed oysters displayed a higher transcriptional activation of these pathways when exposed to POMS which underlies the systemic nature of the microorganisms exposure [23, 28]. Importantly, we identified taxa that were overrepresented in the microbiota of the ME-exposed oysters and which could contribute to improve the better survival capacity that we observed. Among these bacteria, species belonging to the family of *Rhodobacteraceae* have been previously shown to be associated with increased resistance to POMS, and species belonging to Halomonadaceae, Shewanellaceae, and Oceanospirillaceae have been suggested as potential probiotic in aquaculture (ref). Further analysis will be worth to investigate the role of these bacteria for potential applications [18]. Noteworthy, we cannot exclude that the increased in immune competence also relies on stimulation by protists or viruses that our experimental approach did not allow to depict.

Epigenetic mechanisms have recently been recognized as operating at the interface between the microbiota and the host [78–80]. Importantly, the general cellular metabolism is a key player for epigenome modifications. Recent studies have highlighted that derived metabolites from multiple metabolic pathways linked to mitochondrial metabolism and oxidative stress can affect the activity of enzymes involved in histone and DNA methylation and demethylation [81, 82]. Bacterial metabolites such as folate and short-chain fatty acid have already been pinpointed as essential mediators of communication between commensal bacteria and the host through their effects on epigenetic regulatory enzymes [83, 84]. Based on our transcriptional analyses, we clearly observed a metabolic shift in larvae in response to ME exposure, and this trait was inherited by the next generation. We observed that key enzymes involved in glycolysis pathway and TCA cycle were downregulated in response to the microbial exposure. This metabolic shift differs from the Warburg effect which has been shown to be essential for the induction of histone modifications and functional changes necessary for trained immunity in mammals [85, 86]. Interestingly, we observed that enzymes involved in folate synthesis and DNA methylation regulation were downregulated in ME-exposed oysters and their unexposed offspring. Consistent with this observation, we found that the microbial exposure of oyster larvae had an impact on the DNA methylation pattern of the oyster lineage. The most parsimonious explanation for our observations is that the DNA methylation pattern conveys, at least in part, the microbial imprinting that primes the enhanced immune protection that we observed at the intra- and inter-generational level. Some genes related to immune function displayed a differential methylation profile between ME-exposed and control oysters in both generations. However, such changes in methylation level did not necessarily lead to significant changes in expression of the adjacent genes in *cis*. The absence of *cis*-acting association between expression and methylation changes is not unexpected and has been previously reported [87, 88]. The epigenetic code is not universal and results from a complex interplay between several bearers of epigenetic information such as DNA methylation, histone modifications, nuclear spatial remodeling, and ncRNA, which altogether interact to regulate chromatin states. Assaying DNA methylation here was a first attempt to decipher a causal link between the observed innate immune memory and a potential epigenetic imprinting. This absence of causal link raises again the question of the functional role for DNA methylation especially in invertebrates which exclusively harbor gene body methylation. This clearly fuels the current debate on the relationship between DNA methylation and transcription, which is more nuanced than previously appreciated [89]. Nevertheless, and in accordance with previous studies, we found that changes in transcription and DNA methylation occurred in common biological pathways, and overall, we conclude that DNA methylation likely acts together with other epigenetic pathways to actuate long-lasting memory of early life microbial exposure in oysters.

**Fig. 9:**
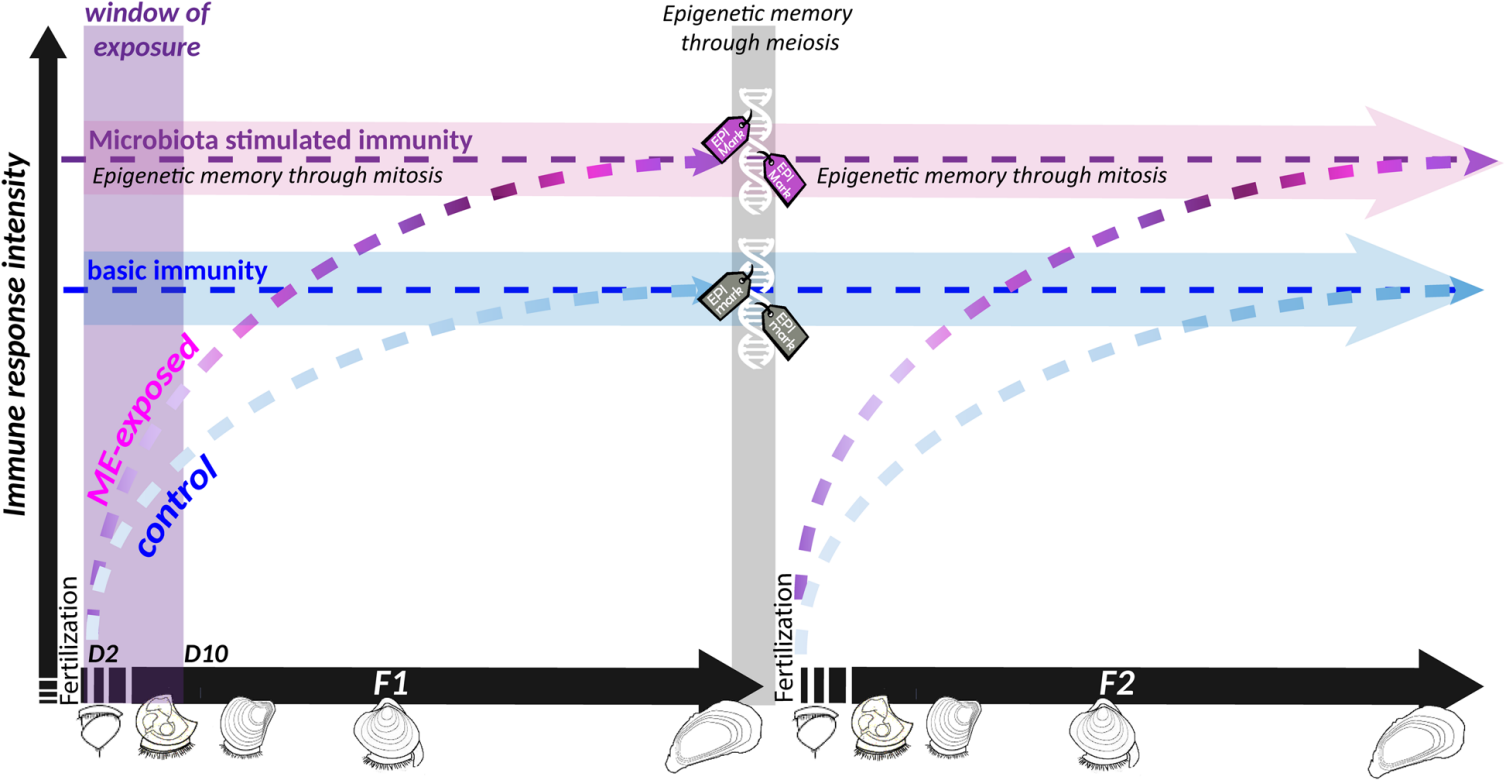
Microbiota-induced epigenetic memory supports lifelong and intergenerational immune protection in *C. gigas*. Schematic representation of the proposed successive events inducing enhanced immune competence in oysters following a microorganism exposure at early stages. Exposing the oyster to microorganism-enriched seawater (ME) increased the diversity and shifted the composition of the oyster microbiota. This change in microbiota during the sensitive window of early development increased the oyster immune competence (symbolized by pink dashed lines) compared to the immune system of control oysters (symbolized by blue dashed lines). Importantly, the enhanced immune system would be expected to exert a pressure on the microflora, resulting in a different bacterial composition in ME-exposed compared to control oysters. The bacterial composition also relies on the developmental stage, which is symbolized by different colors of the dashes. This crosstalk between microbiota and immune system may trigger a continuous reshaping of cellular signaling pathways in host cells which resulted in epigenetic imprinting. This epigenetic memory allowed for inheritance of the phenotype in the offspring of the ME-exposed oysters

In brief, our results support the idea that the rich microbial environment that the oyster’s larvae face just after fertilization in natural seawater is essential to boost the immune system. We showed that this exposure improved the oyster’s immune competence which was maintained across life stages and generations (Fig. 9). Since germ cells develop early during the larval development in *C. gigas* [90], both F1 and F2 generations have experienced the exposure to the ME seawater, either directly during larval stages for the F1 generation or indirectly through germ cell exposure for the F2 generation. In this sense, we report here an intergenerational effect since transgenerational inheritance would require that the change in phenotype is observed in non exposed oysters (including germ cells). We observed a clear DNA methylation change after the microbial exposure, and a large proportion of these epigenetic signatures was heritable. We hypothesize that this inheritance through meiosis may account in part for the intergenerational innate immune memory that we observed, although a direct causal effect remains to be further explored.

## Conclusion

An increasing body of evidence has recently emerged on the microbial instruction of immune education. It has been clearly shown that early life stages are the most appropriate window of opportunity during which the immune system is most sensitive to long-term effects [5, 8]. Here, we were able to successfully increase *C. gigas* immune competence through a natural microbial exposure during larval stages. This biological embedding could be a process applied to the aquaculture context, whereby environmental manipulation through early microbial experience could be used to produce long-lasting resistance to pathogens.

## Supplementary Information

**Additional file 1.** Additional information related to the methodology (the origin of the biological samples, the experimental design for disease induction, the bioinformatic pipelines).

**Additional file 2.** Quality of the metrics for omic analysis (sheet 1: transcriptomic, sheet 2: genetic, sheet 3: epigenomic).

**Additional file 3.** Supplementary results on the phenotypes (Fertilisation success and survival rate of oysters) and on omics data (Table 2 = genetic, Table 3 = transcriptomic, Table 4 = epigenomic, Figs. 1 and 2 = 16S barcoding).

**Additional file 4.** OTU tables for 16S barcoding analysis. Each sheet correspond to a different time point. This sheet also includes the results of LEfSe analysis and compiles the taxa which are statistically significantly enriched in the microbial community of ME-exposed or control oysters in larvae (sheet= LEfSe larval stages) and juveniles (sheet = LEfSe juvenile stages) based on a Kruskal-Wallis test *p*val<0.05.

**Additional file 5.** Results of two differential analyses comparing the proportion of OTUs: (1) ME exposed oysters *vs* control oysters and (2) ME water *vs.* control water.

**Additional file 6.** Compilation of differentially expressed genes at the time point indicated (All DEGs = all the differentially expressed genes, Other sheets = Immune related genes).

**Additional file 7.** Compilation of the RBGOA biological function which are significantly enriched in the transcriptomic dataset for each time point.

**Additional file 8.** Compilation the differentially expressed genes related to metabolic functions at the time point indicated.

**Additional file 9.** Compilation of differentially methylated regions in any regions of the genomes (DMRs), within gene bodies (DMGs), within promoter regions (DMPs).

**Additional file 10.** Compilation the RBGOA biological functions which are significantly enriched in the epigenomic dataset for each time point (sheet 1 to 4) and comparison with RBGOA function from the transcriptomic data.

**Additional file 11.** Details for the statistical analysis performed to adress the significance of the inheritence in DMRs patterns.

## Data Availability

The datasets generated, analyzed during the current study, and supporting the conclusions of this article (RNA-seq, WGBS, WGS, 16S barcoding data) are available at the following link: https://dataview.ncbi.nlm.nih.gov/object/PRJNA609264?reviewer=hg0ttfia8ig7ae6tbam1ssapv8.

## Abbreviations

POMS: Pacific oyster mortality syndrome
UV: Ultraviolet
ME: Microorganism- enriched
NSI: Naissains Standardisés Ifremer
CFU: Colony-forming unit
OTU: Operational taxonomic unit
RNA: Ribonucleic acid
RNA-Seq: Sequencing of the total ribonucleic acid
mRNA: Ribonucleic acid messenger
ncRNA: Noncoding ribonucleic acid
DNA: Deoxyribonucleic acid
gDNA: Genomic deoxyribonucleic acid
DNA-Seq: Ribonucleic acid of the total deoxyribonucleic acid
WGS: Whole genome sequencing
WGBS: Whole genome bisulfite sequencing
BS-seq: Bisulfite sequencing
RBGOA: Rank-based gene ontology analysis
AMPs: Antimicrobial peptides
PCA: Principal component analyses
SNP: Single-nucleotide polymorphism
FLK: Statistical method using an extension of Lewontin and Krakauer (LK) test
FDR: False discovery rate
CpG: Cytosine-phosphodiester bond-guanosine
DEG: Differentially expressed gene
DMR: Differentially methylated region
DMG: Differentially methylated gene
DMP: Differentially methylated promoter
MeV: Multiple Experiment Viewer
ANOVA: Analysis of variance
