# Supplementary material for: Early life microbial exposures shape the Crassostrea gigas immune system for lifelong and intergenerational disease protection
Source: Microbiome. 2022 Jun 4;10:85. doi: 10.1186/s40168-022-01280-5 (PMC9167547; doi:10.1186/s40168-022-01280-5)
Supplement: Supplementary file 2 — Additional file 1. Additional information related to the methodology (the origin of the biological samples, the experimental design for disease induction, the bioinformatic pipelines). [file 40168_2022_1280_MOESM1_ESM.docx]

**Additional file 1 table 1: Number of genitors (males and females) by generation and corresponding fecundation rate.**

| Generation - Family | Numbers of genitors (males/ females) | Fecundation rate (%) |
| --- | --- | --- |
| F0 | 1/1 | 100 |
| F1 | 20/80 | 75 |
| F2 – Exposed | 27/53 | 98 |
| F2 – Control | 29/50 | 96 |

**Additional file 1 table 2: Sampling during the 2 generations experiment**

Sampling was realised in order to perform RNA-Seq, 16S barcoding, genetic and epigenetic analysis on oysters and 16S barcoding analysis on water. Samples were collected during the larval to juveniles’ development (time points indicated in days post fertilisation) or during experimental disease induction (time points indicated at day 120 in Hours post disease induction). Disease induction was performed on day 120 juvenile oysters, Hours 0 represents the time when the donors of pathogens were introduced into the tanks to transmit the disease to the recipient oysters. D120H0 is the juvenile point sampled just before disease induction. D120H3, D120H6 D120H12 is the juvenile point sampled 3 hours, 6 hours and 12 hours after disease induction respectively. For oysters which were sampled during dat day 2 and day 10 of larval stages (D2 and D10 respectively), each sampling point contained 3 pools of 10000 to 20000 larvae. For oysters which were sampled during juvenile’s stages each sampling point contained 3 pools of 10 oysters (day 58 and day 120).

| Generation | Molecular analysis | Developmental time points (Days) | Experimental disease time points (Day120 - Hour) |
| --- | --- | --- | --- |
| Water | Barcoding | D2 |  |
| F0 | Genetic | Gametes from F0 |  |
| F1 | RNA-seq | D2/D10/D120H0 | D120H6/H12 |
|  | Barcoding | D2/D10/D58/D120H0 |  |
|  | Genetic | D120H0 |  |
|  | BS-seq | D10/D120H0 |  |
| F2 | RNA-seq | D10/D120H0 | D120H3/H6/H12 |
|  | Barcoding | D2/D10/D58/D120H0 |  |
|  | Genetic | D120H0 |  |
|  | BS-seq | D10/D120H0 |  |

**Additional file 1 table 3: Experimental and field infection information**

1. Information about recipient’s oysters for each experimental disease induction. The column “Age” represents the age of oysters at the beginning of disease induction (DI).

| Generation | Number of recipient oysters by family and condition | | Weight | Start of disease induction | End of disease monitoring | Age of recipient oysters |
| --- | --- | --- | --- | --- | --- | --- |
|  | Mortalities monitoring | Sampling |  |  |  |  |
| F1 (2016) | 200*2 | 400 | 1400g | 04/07/2016 | +421h | Day 111 |
| F2 (2017) | 100*2 | 320 | 1000g | 05/07/2017 | +320h | Day 97 |

1. Information about donor oysters for each experimental disease induction

| Origin | Cohort number | Total weight |
| --- | --- | --- |
| NSI | 01/2016 | 1800g |
| NSI | 02/2017 | 1800g |

**Additional file 1 figure 1: Experimental design for the disease induction**

Experimental disease induction was performed using pathogen-free donor oysters which were initially placed in a natural environment during an infectious period (temperature >16°C and mortalities recorded in the field) to obtain a microbial community containing the POMS pathogen. These diseased donor oysters (green oysters) were brought back to the laboratory and placed in the same tanks as the recipient oysters. The recipient oysters were 120 days juveniles from the Fa.32 which were either exposed to ME seawater at the F1 generation (ME-exposed oysters, pink oysters) or which were placed in control seawater (Control oysters, blue oysters).

**
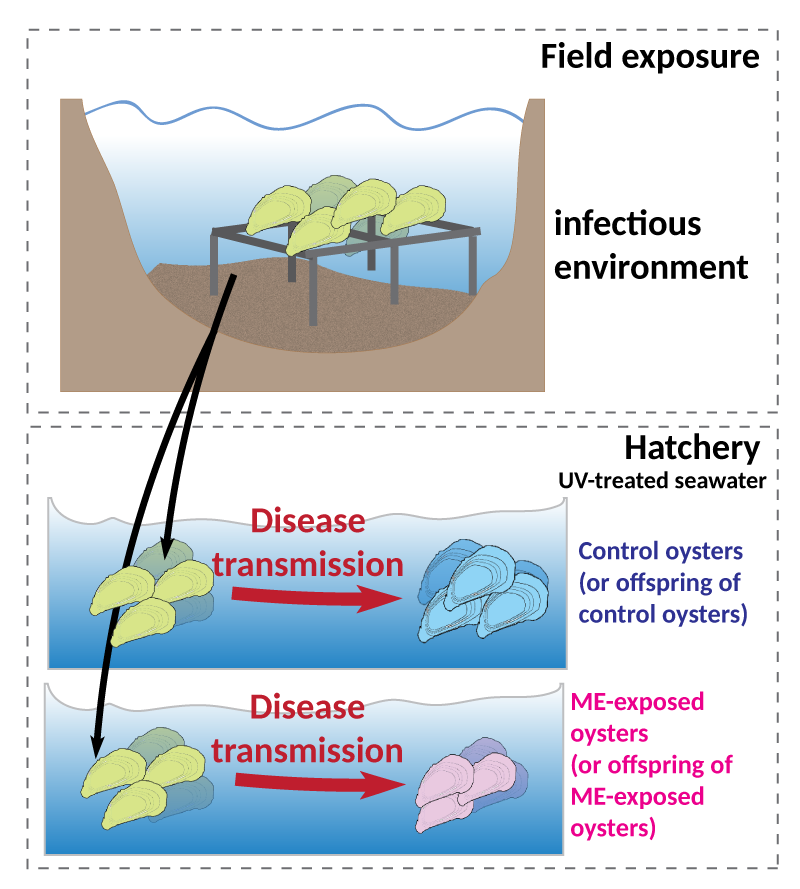
**

**Additional file 1 figure 2: Bioinformatic pipeline for 16S barcoding analysis**

The FROGS pipeline (Find Rapidly OTU with Galaxy Solution) [1] implemented on a galaxy instance [<https://sigenae-workbench.toulouse.inra.fr/galaxy/>] was used for data processing. Paired reads were merged with an authorized mismatch of 0.1 using VSEARCH [2], then primers were removed with cutadapt [3] allowing a 10% differences in the primer search. Sequences clustering was done using SWARM [4] with denoising and aggregation distance d = 3. Chimeras were removed using VSEARCH [2]. After singletons filtering, affiliation was performed using Blast+ against the Silva 132 16S database. Only sequences affiliated to the phylum Bacteria were kept for further analyses.

Community analysis was performed on R software (R Core Team, 2013) using the phyloseq package [5]. Rarefaction curves of species richness were produced using the rarefy-even-depth and ggrare functions [5].

**
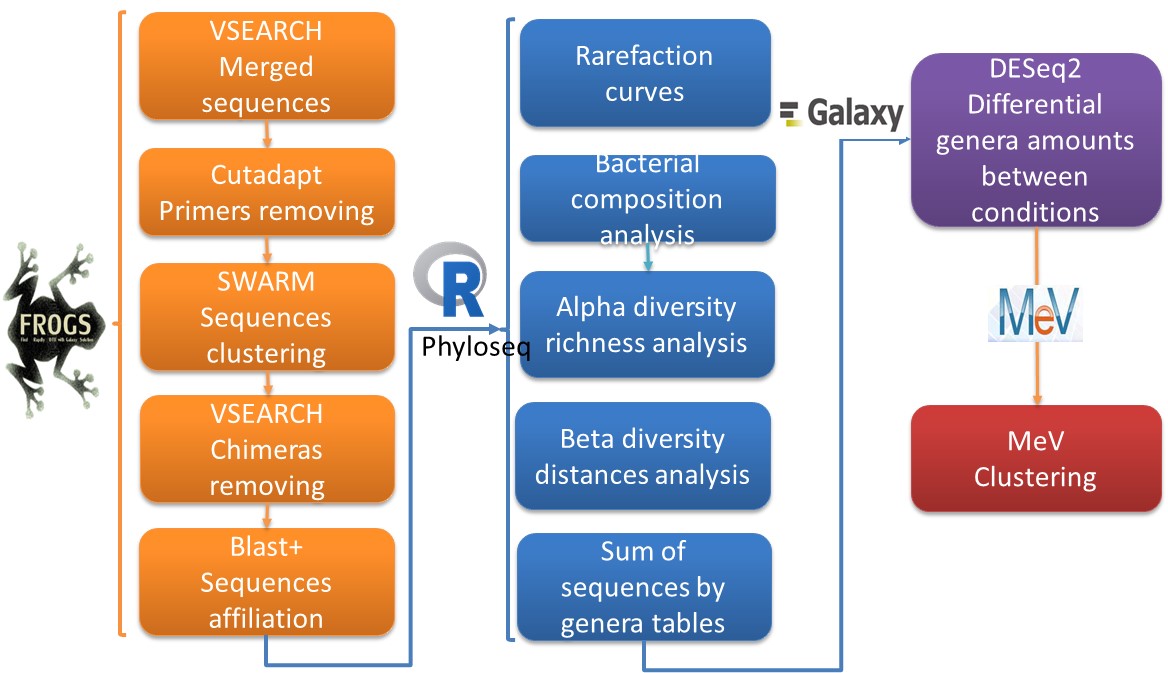
**

**Additional file 1 figure 3: Bioinformatic pipeline for RNAseq analysis**

All data treatments were carried out under a local galaxy instance (http://bioinfo.univ-perp.fr) [6]. Quality of reads were controlled using FastQ Read Quality reports (Galaxy Version 0.70). Phred scores were higher than 26 for more than 90% of the reads length for all the sequences. All the reads were thus kept for subsequent analyses. Reads were mapped on *C. gigas* genome (assembly version V9, [7]) using RNAstar with defaut parameters except for the maximum intron size of 40 000 [8] (Galaxy Version 2.4.0d-2). HTSeq-count was used to count the number of reads overlapping annotated genes (mode Union) (Galaxy Version v0.6.1) [9]. Differential gene expression analysis was performed using DESeq2 [10] (Galaxy Version 2.11.39).

**
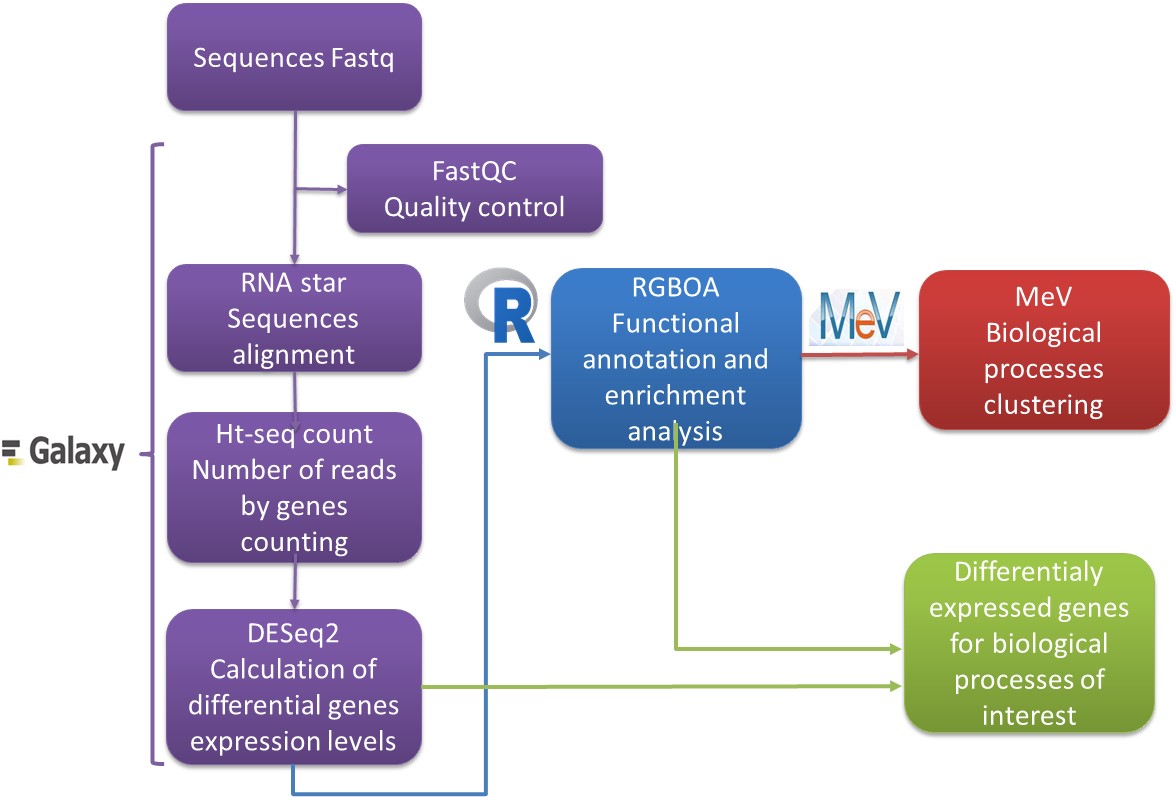
**

**Additional file 1 figure 4: Bioinformatic pipeline for genetic analysis**

Data treatments were carried out under a local galaxy instance [<https://sigenae-workbench.toulouse.inra.fr/galaxy/>] [6]. Reads quality was checked with FastQC with standard parameters (Galaxy Version 0.72, Andrews, 2014). Adapters were removed using TrimGalore, paired-end library, automatic detection of adapter sequences, false Trims 1 bp off every read from its 3’ end, other parameters using as default (Galaxy Version 0.2.8.1, <https://github.com/FelixKrueger/TrimGalore>). Mapping to the *C. gigas* reference genome (assembly version V9, [7] ) was performed using Bowtie 2 with the following parameters: paired-end library, no tweak input options, no tweak alignment options, no tweak scoring options, no use of –a or –k options, no tweak effort options, no tweak SAM/BAM options, no tweak other options, filter unique reads = True (Galaxy Version 2.2.6.2, [11, 12]). BAM files were coordinate sorted with SAMTools. RmDup of SAMtools (paired-end data, treat as single-end = false) was used in order to remove PCR duplicates (Galaxy Version 1.0.0, [13]). MPileup was used to generate VCF files necessary for the following analysis and containing key information for each genomic position. The following parameters were used: do not perform genotype likelihood computation, output base positions on reads = false, output mapping quality = false, filter by flags to exclude or require, exclude = the read is a PCR or optical duplicate, do not limit select regions to call, do not exclude select read groups, do not disable read-pairs overlap detection, skip anomalous read pairs in variant calling, disable probabilistic realignment for the computation of base alignment quality, coefficient for downgrading mapping quality for reads containing excessive mismatches = 0, max reads per BAM = 250, do not redo BAQ computation, minimum mapping quality for an alignment to be used = 20, minimum base quality for a base to be considered = 13, only generate pileup in region = empty (Galaxy Version 0.0.1, [14]). Within each sample, allelic frequencies were estimated using Pool-HMM [15] for all genomic positions that were covered by at least five reads (option-c). One prerequisite for this estimation step is to compute the genome-wide allele frequency spectrum; for this task, the proportion of genomic positions used was set to 0.001 (option −R) according to the author's recommendations, and the starting value for the population mutation rate (option −t) was the default value 0.005. Allele frequency files obtained for each sample were then merged using the python script estim2freq.py, available from the pool‐hmm webpage (<https://forge-dga.jouy.inra.fr/projects/pool-hmm>). Only polymorphic positions with a minor allele frequency (computed over all samples) greater than 5% were kept at this step.

**
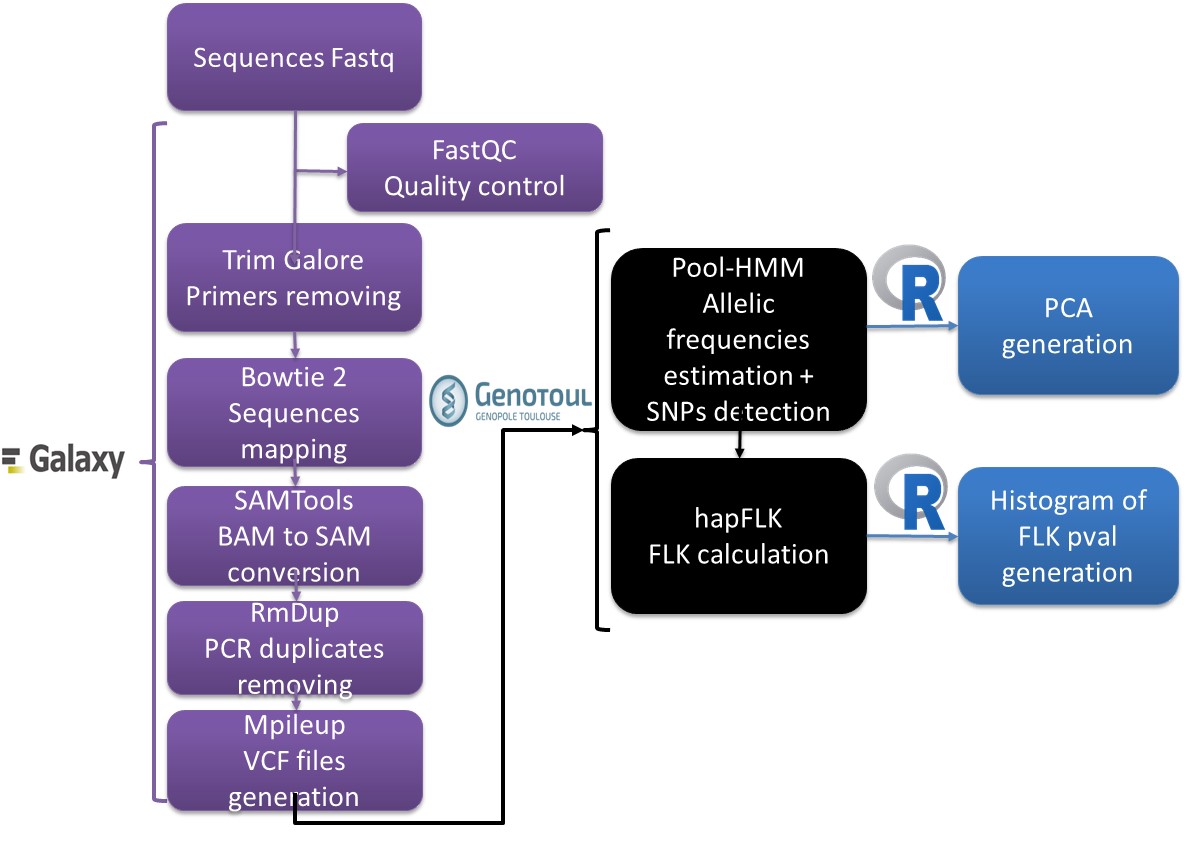
**

**Additional file 1 figure 5: Bioinformatic pipeline for DMRseq analysis**

Reads quality was checked using FastQC. Nucleotide positions with a quality score below 20 were trimmed using Trimgalore (paired-end, both strands, phred quality score = 20). Reads were then aligned on the *C. gigas* reference genome (assembly version V9, [7]) with Bismark [16] using the following parameters: maximum insert size = 500, no mismatch allowed, sort Bam file by chromosomal position, other parameters used as default). Clustering of the data was performed from BAM files using MethylKit [17]. PCA analysis was performed with R based on 50000 randomly picked CpG positions. Using MethylExtract [18] and the BAM files from Bismark, the methylation level of each cytosines was synthesised in different file formats (Bed, Wig and VCF). The Wig files were loaded on Integrative Genomics Viewer (IGV) for visual inspection of the data [19]. Differential methylation analyses were performed with DMRseq package using the following parameters blocksize= TRUE, minnumregion=3, deltamax=0.25, bpspan=1000, mininspan=10, maxgapssmooth=2500, smooth=TRUE [20].

**
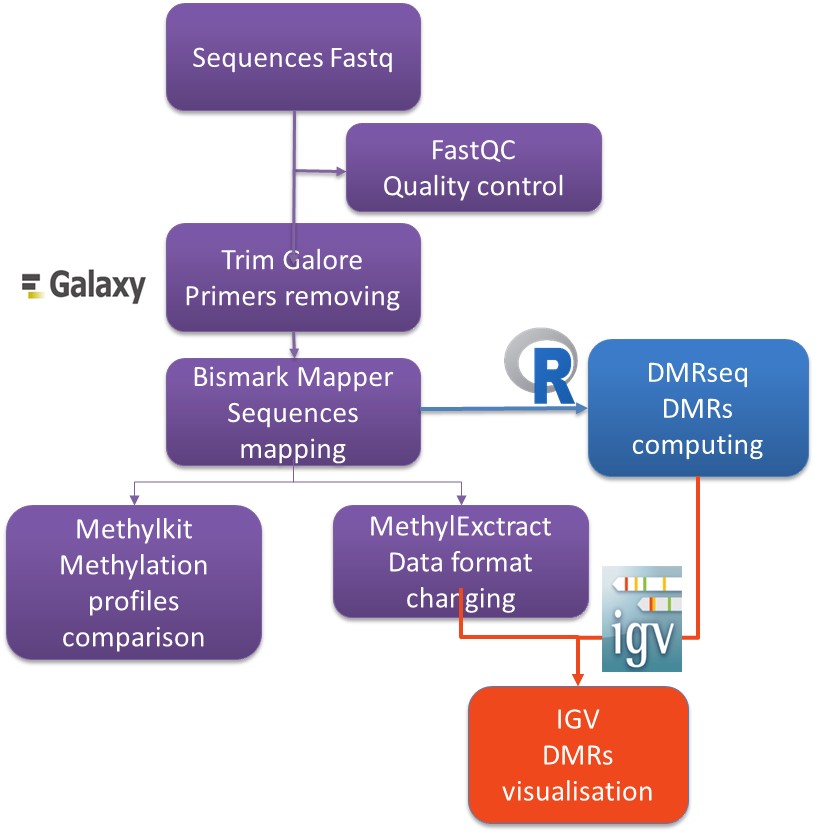
**

**References**

1. Escudie F, Auer L, Bernard M, Mariadassou M, Cauquil L, Vidal K, Maman S, Hernandez-Raquet G, Combes S, Pascal G: FROGS: Find, Rapidly, OTUs with Galaxy Solution. *Bioinformatics* 2018, 34(8):1287-1294.

2. Rognes T, Flouri T, Nichols B, Quince C, Mahe F: VSEARCH: a versatile open source tool for metagenomics. *PeerJ* 2016, 4:e2584.

3. Martin M: Cutadapt removes adapter sequences from high-throughput sequencing reads. *EMBnetjournal* 2011, 17:10-12.

4. Mahe F, Rognes T, Quince C, de Vargas C, Dunthorn M: Swarm: robust and fast clustering method for amplicon-based studies. *PeerJ* 2014, 2:e593.

5. McMurdie PJ, Holmes S: phyloseq: an R package for reproducible interactive analysis and graphics of microbiome census data. *Plos One* 2013, 8(4):e61217.

6. Goecks J, Nekrutenko A, Taylor J: Galaxy: a comprehensive approach for supporting accessible, reproducible, and transparent computational research in the life sciences. *Genome Biology* 2010, 11(8):R86.

7. Zhang G, Fang X, Guo X, Li L, Luo R, Xu F, Yang P, Zhang L, Wang X, Qi H *et al*: The oyster genome reveals stress adaptation and complexity of shell formation. *Nature* 2012, 490(7418):49-54.

8. Dobin A, Davis CA, Schlesinger F, Drenkow J, Zaleski C, Jha S, Batut P, Chaisson M, Gingeras TR: STAR: ultrafast universal RNA-seq aligner. *Bioinformatics* 2013, 29(1):15-21.

9. Anders S, Pyl PT, Huber W: HTSeq--a Python framework to work with high-throughput sequencing data. *Bioinformatics* 2014, 31(2):166-169.

10. Love MI, Huber W, Anders S: Moderated estimation of fold change and dispersion for RNA-seq data with DESeq2. *Genome Biology* 2014, 15(12).

11. Langmead B, Trapnell C, Pop M, Salzberg SL: Ultrafast and memory-efficient alignment of short DNA sequences to the human genome. *Genome Biology* 2009, 10(3).

12. Langmead B, Salzberg SL: Fast gapped-read alignment with Bowtie 2. *Nat Methods* 2012, 9(4):357-359.

13. Li H, Handsaker B, Wysoker A, Fennell T, Ruan J, Homer N, Marth G, Abecasis G, Durbin R: The Sequence Alignment/Map format and SAMtools. *Bioinformatics* 2009, 25(16):2078-2079.

14. Li H: A statistical framework for SNP calling, mutation discovery, association mapping and population genetical parameter estimation from sequencing data. *Bioinformatics* 2011, 27(21):2987-2993.

15. Boitard S, Kofler R, Francoise P, Robelin D, Schlotterer C, Futschik A: Pool-hmm: a Python program for estimating the allele frequency spectrum and detecting selective sweeps from next generation sequencing of pooled samples. *Molecular ecology resources* 2013, 13(2):337-340.

16. Krueger F, Andrews SR: Bismark: a flexible aligner and methylation caller for Bisulfite-Seq applications. *Bioinformatics* 2011, 27(11):1571-1572.

17. Akalin A, Kormaksson M, Li S, Garrett-Bakelman FE, Figueroa ME, Melnick A, Mason CE: methylKit: a comprehensive R package for the analysis of genome-wide DNA methylation profiles. *Genome Biology* 2012, 13(10):R87.

18. Barturen G, Rueda A, Oliver JL, Hackenberg M: MethylExtract: High-Quality methylation maps and SNV calling from whole genome bisulfite sequencing data. *F1000Research* 2013, 2:217.

19. Thorvaldsdottir H, Robinson JT, Mesirov JP: Integrative Genomics Viewer (IGV): high-performance genomics data visualization and exploration. *Briefings in bioinformatics* 2013, 14(2):178-192.

20. Korthauer K, Chakraborty S, Benjamini Y, Irizarry R: Detection and accurate false discovery rate control of differentially methylated regions from whole genome bisulfite sequencing. *Biostatistics* 2018, 20:367-383.
