# Supplementary material for: Early life microbial exposures shape the Crassostrea gigas immune system for lifelong and intergenerational disease protection
Source: Microbiome. 2022 Jun 4;10:85. doi: 10.1186/s40168-022-01280-5 (PMC9167547; doi:10.1186/s40168-022-01280-5)
Supplement: Supplementary file 4 — Additional file 3. Supplementary results on the phenotypes (Fertilisation success and survival rate of oysters) and on omics data (Table 2 = genetic, Table 3 = transcriptomic, Table 4 = epigenomic, Figs. 1 and 2 = 16S barcoding). [file 40168_2022_1280_MOESM3_ESM.docx]

**Additional file 3 table 1: Fertilisation success and survival rate during oyster development in the F1 generation**

The number of larvae was counted at day 2 (D larvae) and juvenile oysters at day 120, and the developmental rate was calculated compared to total number of fertilised gametes introduced in each pipe. The mean and standard deviation of the developmental rate in the three pipes is indicated for day 2. No statistically different developmental rates were recorded between ME-exposed and control oysters. Note that at day 120 only one count could be performed since all the oysters were gathered into a unique tank. Day 0 data represent the fertilisation rate.

|  |  | Day 0 | Day 2 | Day 120 |
| --- | --- | --- | --- | --- |
| F1 | **Control**  **ME-exposed** | 75.0  75.0 | 58.2 ± 4.8  54.7 ± 5.7 | 2.1  2.8 |

**Additional file 3 table 2: Number of significant SNPs between ME-exposed and control oysters**

SNPs were called at a False Discovery Rate (FDR) of 5%, 10%, 15% and 20% following the Storey et al. (2003) study [1].

| **Generations** | **Number of significant SNPs (FDR<0.05)** | **Number of significant SNPs (FDR<0.1)** | **Number of significant SNPs (FDR<0.15)** | **Number of significant SNPs (FDR<0.2)** |
| --- | --- | --- | --- | --- |
| **F1** | 0 | 7 765 | 7 827 | 7 836 |
| **F2** | 0 | 0 | 0 | 8 441 |

**Additional file 3 table 3: Differentially Expressed Genes in ME-exposed and control oysters**

Genes statistically significantly differentially expressed (DEGs, adjusted *p-val* < 0.05) were determined using DeSEq2 analysis out of the 28 027 genes of the *C. gigas* reference genome V9 [2]. Comparison was performed between the ME-exposed and control oysters for the following time points of the F1 generation: Day2, 10 and Day 120 = Hour 0, 6 12 after disease induction and for the following time point of the F2 generation: Day 10, Day 120 = Hour 0, 3, 6, 12 after disease induction. Each DEG is reported in the additional file 6.

|  |  | Differentially Expressed Genes | Induced | Repressed |
| --- | --- | --- | --- | --- |
| F1 | Day 2 | 3 410 | 1 311 | 2 098 |
|  | Day 10 | 1 100 | 537 | 563 |
|  | Day 120 Hour 0 | 35 | 21 | 14 |
|  | Day 120 Hour 6 | 80 | 23 | 57 |
|  | Day 120 Hour 12 | 53 | 26 | 27 |
|  |  |  |  |  |
| F2 | Day 10 | 6 029 | 2 845 | 3 184 |
|  | Day 120 Hour 0 | 120 | 58 | 62 |
|  | Day 120 Hour 3 | 249 | 165 | 84 |
|  | Day 120 Hour 6 | 67 | 49 | 18 |
|  | Day 120 Hour 12 | 104 | 56 | 48 |

**Additional file 3 table 4: Differentially Methylated Regions between ME-exposed and control oysters**

Regions statistically differentially methylated (DMRs) were determined using the DMRSeq package and based on a *p-val* < 0.05 among the 28 027 genes of the *C. gigas* reference genome V9^2^. Comparison was performed between the ME-exposed and control oysters for the following time point of the F1 and F2 generation: Day10 and Day 120 Hour 0 (just before disease induction). Each DMRs is reported in the additional file 9.

| Generation | Time point | Total number of DMRs | Number and % of hyper-methylated DMRs | | Number and % of hypo-methylated DMRs | |
| --- | --- | --- | --- | --- | --- | --- |
| F1 | Day 10 | 4325 | 2482 | 57.4% | 1843 | 42.6% |
|  | Day 120 | 4985 | 2040 | 40.9% | 2945 | 59.1% |
| F2 | Day 10 | 5531 | 1230 | 22.2% | 4301 | 77.8% |
|  | Day 120 | 5207 | 2550 | 49.0% | 2657 | 51.0% |

**Additional file 3 figure 1: Microorganism-Enriched seawater exposure alters the bacterial diversity of oyster microbiota**

(a) Chao1 index (y-axis), (b) Shannon index (y-axis) and (c) Simpson index (y-axis) from 16S barcoding data obtained from oysters sampled at day 2 for ME-exposed (pink) and control (blue) oysters. * represents a statistically significant different index in the ME-exposed compared to the control oysters *i.e*: ANOVA, *p-val* < 0.05.

**
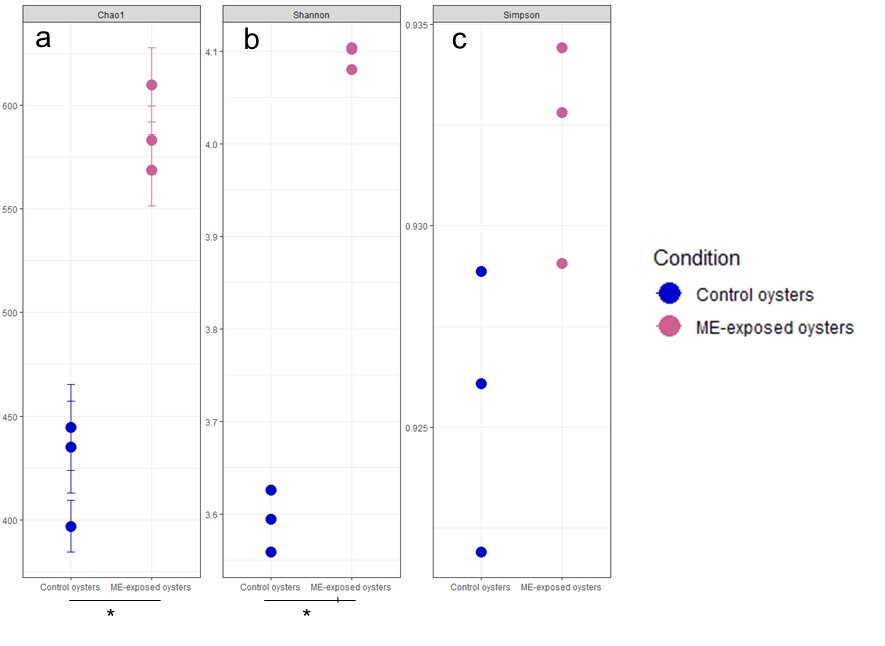
**

**Additional file 3 figure 2: The oyster microbiota changes significantly over the course of development**

Principal coordinate analyses (PCoA) representing dissimilarities between samples using the Bray-Curtis distance matrix performed on 16S barcoding data obtained in oysters sampled at different the developmental stages (day 2, 10, 58, 120 after fertilisation) for generation F1 (a) and F2 (b). Oysters exposed to ME seawater are indicated by purple dots and oysters raised in control seawater are indicated by blue dots. For the F1 and F2 generations, differences between developmental times are significant: multivariate homogeneity of group dispersions: *p-val* = 0,001, pairwise comparison for each: *p-val* = 0,006.


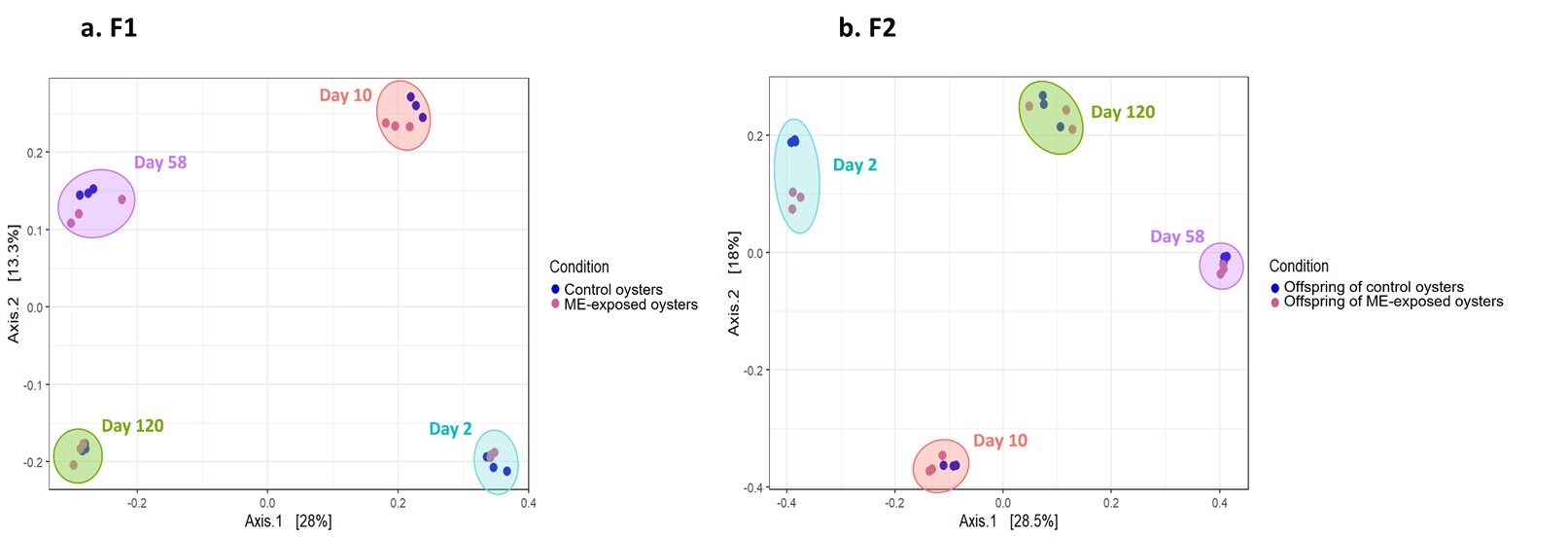


**Additional file 3 figure 3: Specific taxa are overrepresented in ME-exposed or control oysters.**

The LEfSe algorithm was applied to identify bacterial taxa statistically significantly overrepresented in the microbiota of the ME-exposed and control oysters at larval (a) and juvenile (b) stages. The LDA score indicates the ranking of the biomarker relevance (log 10 scale).

**
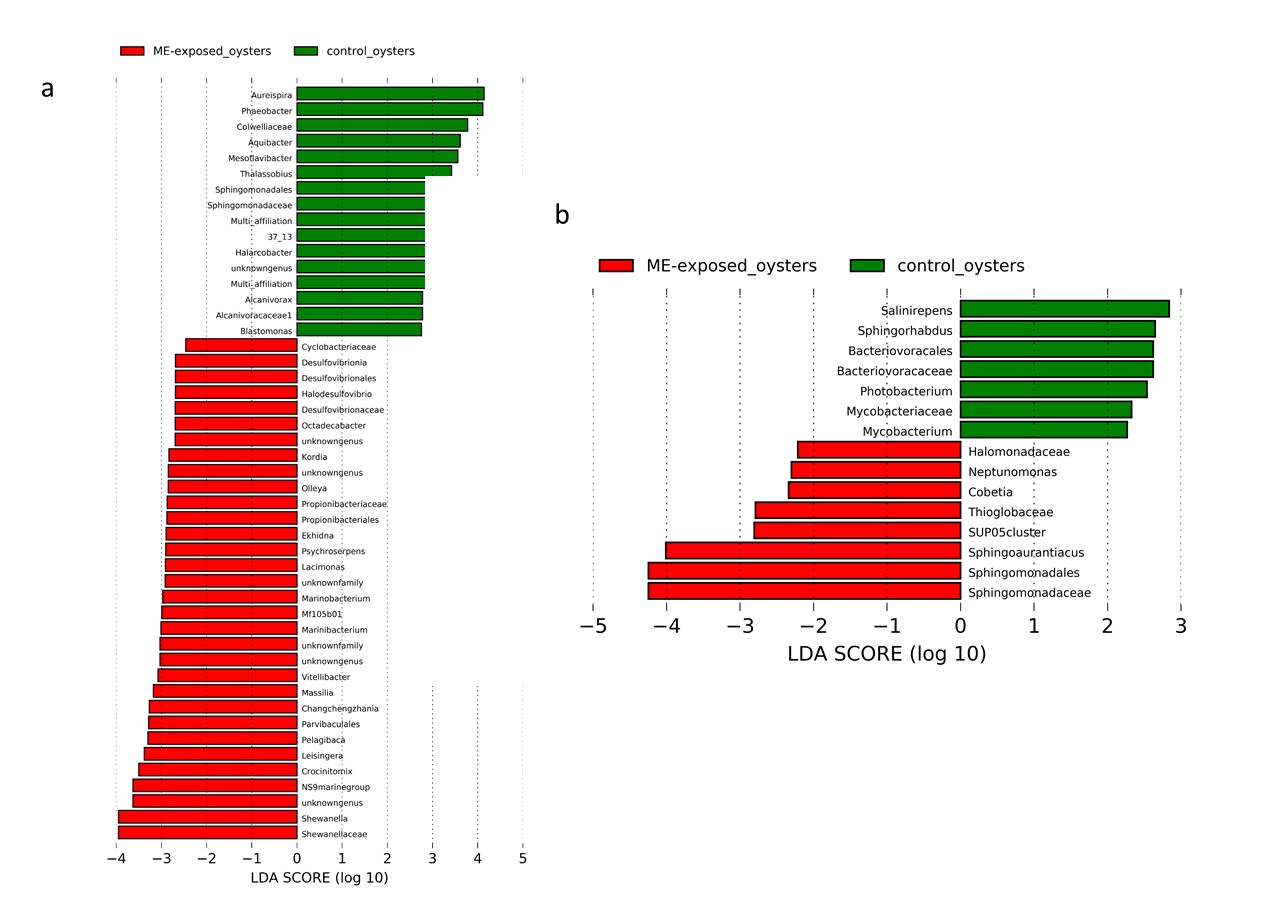
**

**Additional file 3 figure 4: ME-exposed oysters and their offspring displayed enhanced survival when facing POMS in the field**

Survival curves of oysters (y-axis) in the field during POMS disease outbreak (in days = x-axis) in ME-exposed (pink line) and control (blue line) oysters for F1 generation (a) and offspring of ME-exposed or control oysters for F2 generation (b). P-values of log-rank tests and final survival percentages for each condition are indicated on each survival curve.

**
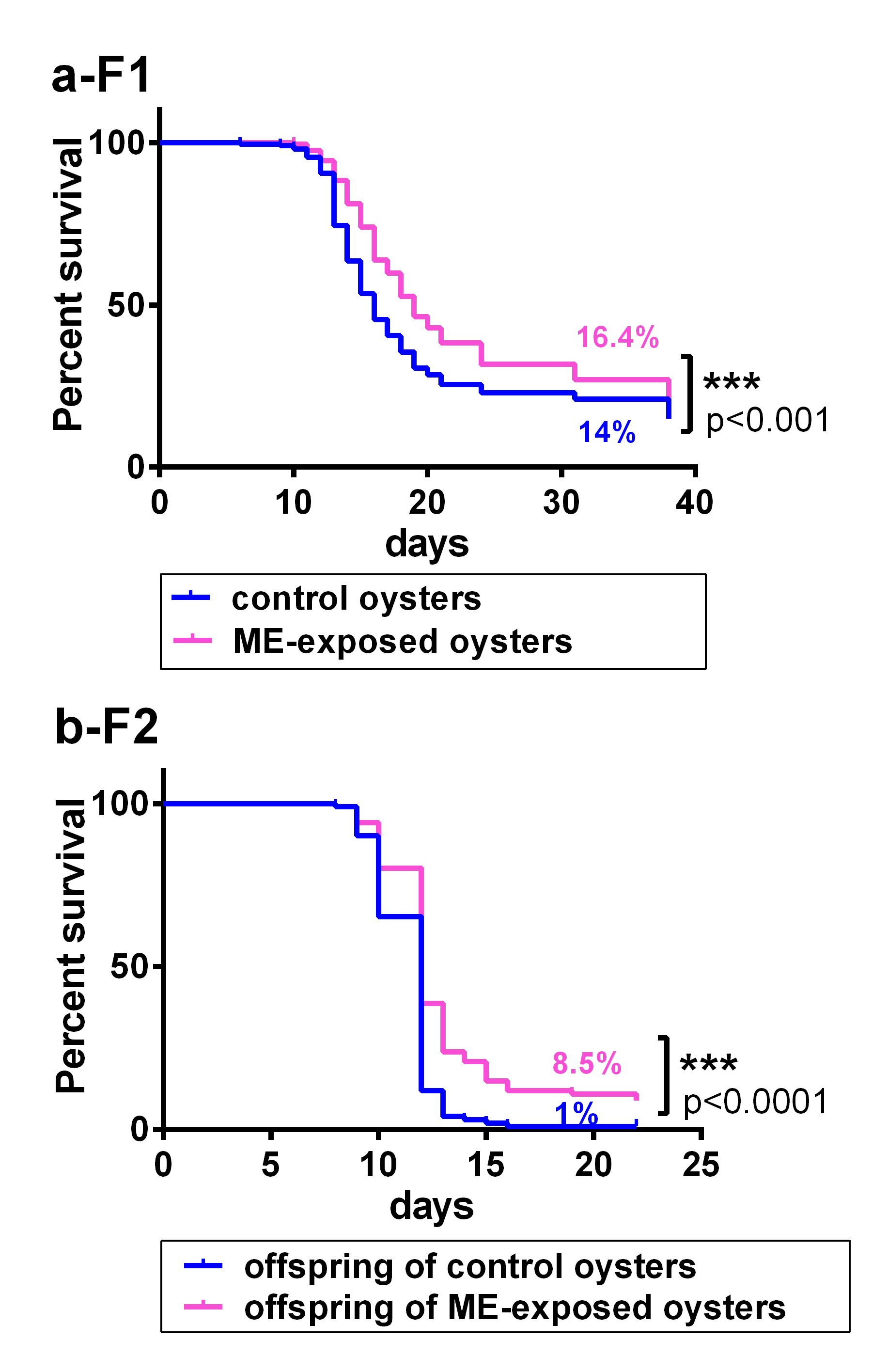
**

**Additional file 3 figure 5:** **CpG methylation patterns clustered according to developmental stages**

A principle coordinate analysis representing the methylation pattern of each sampled oysters based on 50000 randomly picked methylated CpG sites per sample was generated for ME-exposed (EXP) and control (C) oysters of day10 (D10, red color) and and day120 (D120H0, green color) of both generations (F1 and F2) for each of the three biological replicates. For exemple, the sample D120H0_F1_C1 represent the methylation pattern of the replicate 1 of oyster sampled at juvenile stage (Day120) of the F1 generation of the control condition.

**
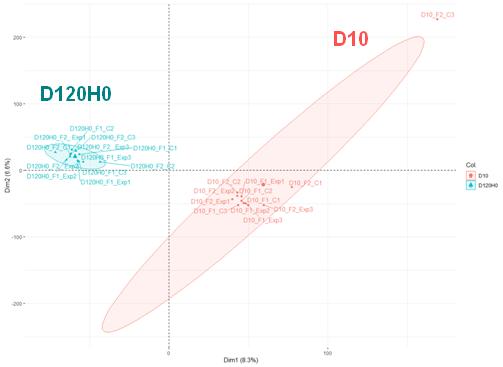
**

**Additional file 3 figure 6:** **Global level of CpG methylation is significantly different between larval and juvenile stages**

Level of CpG methylation as a percentage of all cytosines at day 10 (D10) and day 120 (D120H0) are represented for each condition: ME-exposed (Pink) and control (Blue) oysters and offspring of ME-exposed (Pink) and control (Blue) oysters. Each boxplot represents the repartition of percentage of cytosine methylation of three biological replicates obtained from bismark analysis. The bar represents the median. Statistical differences between ME-exposed and control oysters were tested with Wilcox-test, and no significant difference was found. Statistical differences between developmental stages (Day 10 *vs* Day 120) were tested with Wilcox-test, and a *p-val*<0.01 was obtained for both generations. *** =** *p-val*<0.01.

**
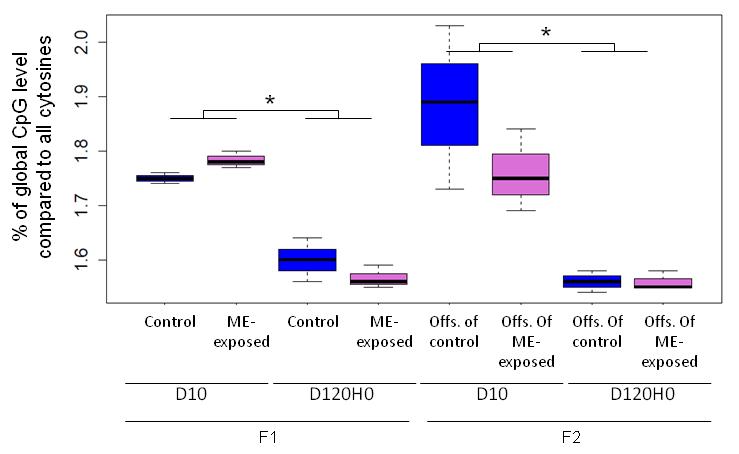
**

**Additional file 3 figure 7: Intragenic DNA methylation changes occurs in genes linked to immune functions**

The heatmap represents genes encoding immune functions which displayed statistically significant differential methylation between ME-exposed and control oysters at day 10 (F1_D10) and at day 120 (F1_D120H0) and between offspring of ME-exposed and offspring of control oysters at day 10 (F2_D10) and at day 120 (F2_D120H0). The heatmap is based on the significant *p-val* obtained in the DMR-seq analysis.


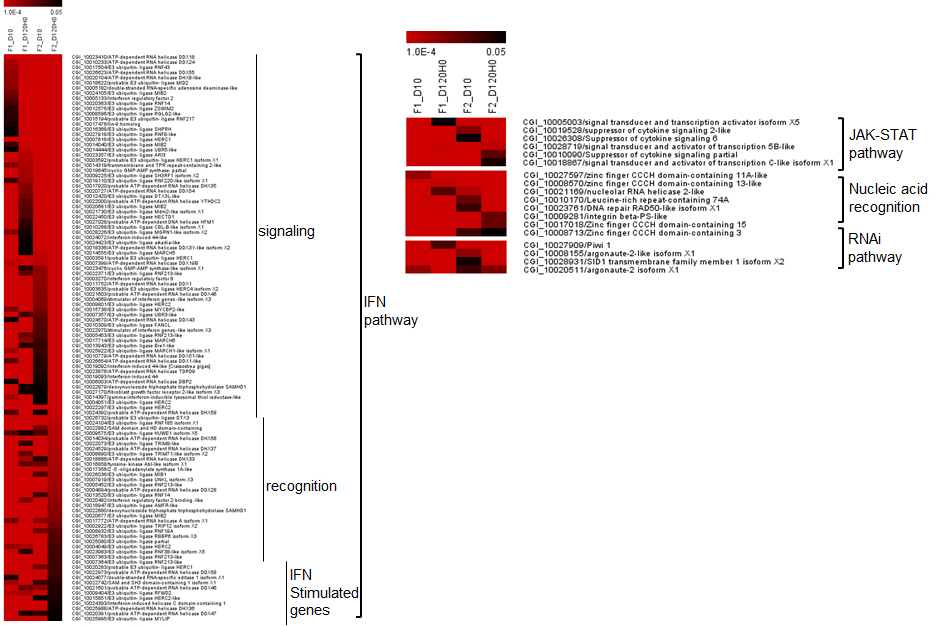


**Additional file 3 figure 8: Hyper- and hypo-methylated DMRs are not biased toward induction or repression of gene expression at day 10 of F2 generation**

The 6029 DEGs (ME-exposed *vs* control oysters for F2 day 10) displayed 47% induced (blue bars) and 53% repressed (orange bars) genes. Among the 540 hypo-methylated and the 200 hyper-methylated DMRs that intersected with these 6029 DEGs (*ie* DMGs), 46% and 54% were induced and repressed genes, respectively (for hypo DMGs) and 44% and 56% were induced and repressed genes (for hyper DMGs), respectively. Among the 136 hypo-methylated and the 33 hyper-methylated DMRs that intersected with the promoter region (ie DMPs) of these 6029 DEGs, 40% and 60% were induced and repressed genes (for hypo DMPs) and 42% and 58% were induced and repressed genes (for hyper DMPs), respectively. These percentages in induced and repressed genes among the hyper and hypo DMRs represent roughly the same percentage obtained in the total 6029 DEGs.


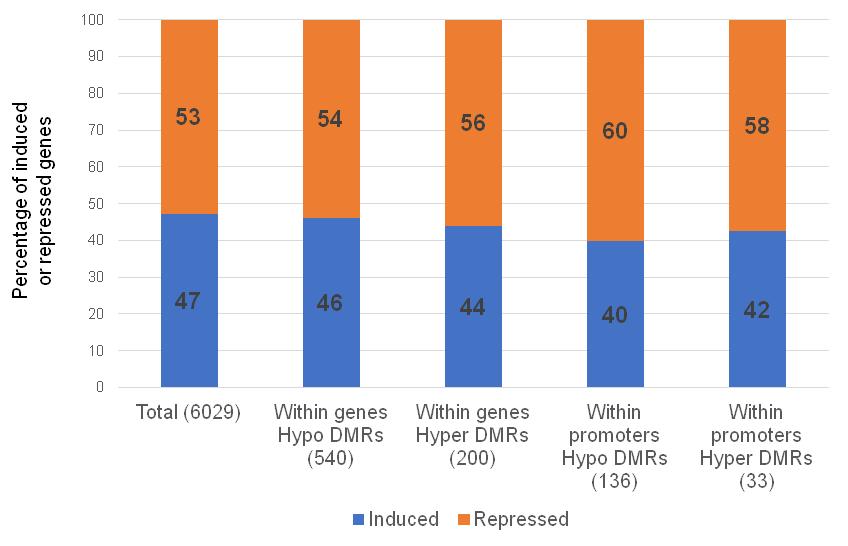


**References**

1. Storey JD, Tibshirani R: Statistical significance for genomewide studies. *P Natl Acad Sci USA* 2003, 100(16):9440-9445.

2. Zhang G, Fang X, Guo X, Li L, Luo R, Xu F, Yang P, Zhang L, Wang X, Qi H *et al*: The oyster genome reveals stress adaptation and complexity of shell formation. *Nature* 2012, 490(7418):49-54.
